# Structures and Biological Activities of Diketopiperazines from Marine Organisms: A Review

**DOI:** 10.3390/md19080403

**Published:** 2021-07-21

**Authors:** Zhiqiang Song, Yage Hou, Qingrong Yang, Xinpeng Li, Shaohua Wu

**Affiliations:** State Key Laboratory for Conservation and Utilization of Bio-Resources in Yunnan, Key Laboratory for Southwest Microbial Diversity of the Ministry of Education, School of Life Sciences, Yunnan Institute of Microbiology, Yunnan University, Kunming 650091, China; songzhiqiang@mail.ynu.edu.cn (Z.S.); houyage@126.com (Y.H.); yangqingrong132@163.com (Q.Y.); lixinpeng10086@126.com (X.L.)

**Keywords:** natural products, chemical structures, diketopiperazines, biological activities

## Abstract

Diketopiperazines are potential structures with extensive biological functions, which have attracted much attention of natural product researchers for a long time. These compounds possess a stable six-membered ring, which is an important pharmacophore. The marine organisms have especially been proven to be a wide source for discovering diketopiperazine derivatives. In recent years, more and more interesting bioactive diketopiperazines had been found from various marine habitats. This review article is focused on the new 2,5-diketopiperazines derived from marine organisms (sponges and microorganisms) reported from the secondary half-year of 2014 to the first half of the year of 2021. We will comment their chemical structures, biological activities and sources. The objective is to assess the merit of these compounds for further study in the field of drug discovery.

## 1. Introduction

The 2,5-diketopiperazines (DKPs), the smallest cyclic dipeptides from the double condensations of two *α*-amino acids, are abundant in nature and possess a six-membered piperazine rigid backbone [1,2]. The formation of two peptide bonds in DKPs are catalyzed by two major enzymes, the nonribosomal peptide synthetases (NRPSs, >100 kDa) and cyclodipeptide synthases (CDPSs, 30 kDa) [3]. These three-dimensional molecular skeletons carry different substituents, which overcome the planar limitations of most conventional drugs and play an important role in drug discovery [4]. Recently, the interest in DKPs is still high because they have not only antimicrobial, antitumor and antiviral activities [5], but also a relatively rare treatment of ischemic brain injury [6], quorum-sensing signaling [7,8], anti-Alzheimer [9], inhibition of microtubule polymerization [10] and haemosuppressor activity [11].

Bicyclomycin is a classic DKP antibiotic that has been used to treat diarrhea in humans and bacterial diarrhea in calves and pigs and it is also a rho (a member of the RecA-type ATPase) inhibitor [12,13,14]. Chaetocin is a specific DKP dimer containing sulfurs as the first inhibitor of a lysine-specific histone methyltransferase SU(VAR)3-9, which could reduce the H3 isoform trimethylated at the Lys9 (H3K9me3) level and this compound has also been reported to have potent antimyeloma activity [15,16,17]. Recently, chaetocin is proved to be able to increase the efficiency of the efficient epigenetic reprogramming via reducing the aberrant level of H3K9me3 to enhance the developmental competence of porcine somatic cell nuclear transfer embryos. It promotes osteogenic differentiation in mesenchymal stem cells [16,18,19]. Plinabulin (formerly named as NPI-2358), a marine-derived DKP, is currently in a phase 3 trial in combination with docetaxel in non-small cell lung cancer (NCT02504489) [20,21,22]. In addition, some DKPs possess an ability to cross the blood–brain barrier via a passive diffusion process as an ideal candidate for new therapeutic agents for brain diseases [23,24]. As of August 2012, there are approximately 150 global patents related to DKPs and its derivatives, and DKPs are present in nearly 50 bio-complexes in the Research Collaboratory for the Structural Bioinformatics Protein Data Bank [25,26]. DKPs are diamonds in the rough and have huge potential in future therapies.

There have been several reviews reported on DKPs until now. Cao et al. summarized chemical diversity and the biological function of indolediketopiperazines from marine-derived fungi [27]. Gomes et al. summed up marine-derived DKP dimers with their structures and biological activity [28]. From 1972 to the first half of the year of 2014, 214 DKPs from marine sources have been reported [5,29]. However, there is no relevant review for summarizing the comprehensive DKPs from a marine source from 2014 to now. On this basis, we now summarize a total of 241 marine-derived DKPs from the second half of 2014 to the first half of 2021 in this paper. In addition, a total of 55 marine-derived variable DKP derivatives from 2011 to the first half of 2021 are also summarized here.

## 2. Chemical Structures of Diketopiperazines from Marine Organisms

The 241 DKPs from different sources including sponges, bacteria, actinomycetes and fungi are shown in Table 1.

### 2.1. Sponge

**Figure 1:**
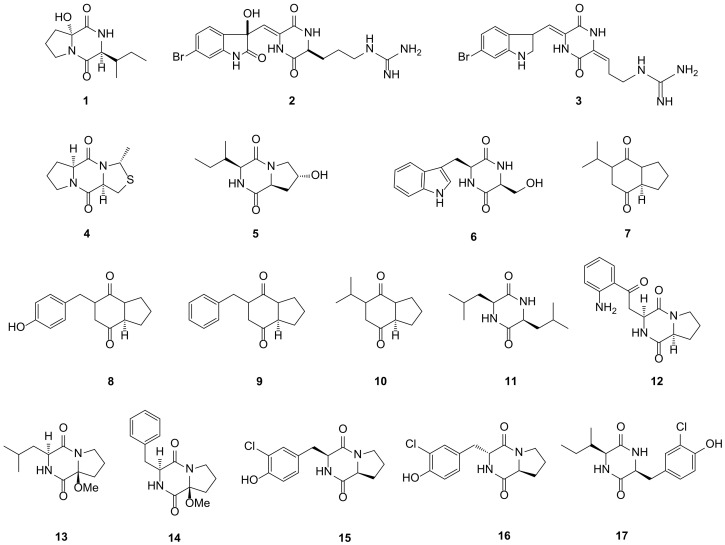
DKP structures from sponge (**1***–***5**) and bacteria (**6***–***17**).

Cyclo-(R-Pro-6-hydroxyl-S-Ile) (**1**) was a new DKP isolated from the sponge *Callyspongia* sp., which was collected from the South China Sea. Compound **1** showed no antibacterial activity against the tested *Bacillus subtilis*, *Staphylococcus aureus* and *Escherichia coli* [30]. Geobarrettins A (**2**) and B (**3**), two new DKPs, were obtained from the sub-Arctic sponge *Geodia barrette,* which was collected at the west of Iceland (-388 m). Before coculturing with allogeneic CD4^+^ T cells, the maturing human dendritic cells (DCs) were processed by compound **3** with a dose of 10 μg/mL and then reduced the IFN-*γ* of T cell secretion. Compound **3** had no effect on the DCs secretion of IL-10 but induced the IL-12p40. This above data demonstrated that compound **3** possessed an overall anti-inflammatory activity and may be used to treat the Th1 type inflammation [31]. A novel sulfur-containing DKP, tedanizaine A (**4**), was collected from the marine sponge *Tedania* sp. at a depth of 10 m in Zhanjiang, Guangdong province. Compound **4** bearing a thiazolidine unit was separated by integrating molecular networking and became the second example of thiodiketopiperazine. However, the evaluation of cytotoxicity activities did not reveal the inhibitory activity of compound **4** on the growth of the tested A549 (lung carcinoma) and RAW 246.7 (macrophage) cell lines [32]. (−)-Cyclo(L-trans-Hyp-L-Ile) (**5**) was a new DKP isolated from the marine sponge *Axinella sinoxea*, collected from a reef habitat around Larak Island, Persian Gulf. Compound **5** had no influence on methicillin-resistant *Staphylococcus aureus* (MRSA) in the dose of 100 μg/mL [33]. All 5 DKPs from sponge described above are presented in Figure 1.

### 2.2. Bacteria

Cyclo (Trp-Ser) (**6**) was a novel DKP from *Rheinheimera aquimaris* QSI02, in the Yellow Sea of Qingdao, which was active against *E*. *coli*, *Chromobacterium violaceum* CV026 and *Pseudomonas aeruginosa* PA01 with the minimum inhibitory concentration (MIC) values of 6.4, 3.2 and 6.4 mg/mL, respectively. Compound **6** possessed antiquorum sensing activity, which could decrease the QS-regulated violacein and pyocyanin production by 67% and 65% in *C. violaceum* CV026 and *P. aeruginosa*. Based on the molecular docking results, compared with the natural signaling molecule, compound **6** was easier to combine with the CviR receptor, but opposite in the LasR receptor. These consequences indicated that compound **6** may become a potential inhibitor to control the quorum sensing (QS) system [34]. Four novel DKPs were isolated from psychrophilic yeast *Glaciozyma antarctica* PI12, which was collected from a marine environment in Antarctica, and they were named as cyclo(Pro-Val) (**7**), (−)-cyclo(Pro-Tyr) (**8**), (−)-cyclo(Pro-Phe) (**9**) and (+)-cyclo(Pro-Leu) (**10**). However, all these compounds reported in the present study were not subjected to further bioactivity studies [35]. (3*S*,6*S*)-3,6-diisobutylpiperazine-2,5-dione (**11**) was firstly displayed from a sponge-associated bacterium *Bacillus* sp. SPB7, which was isolated from the sponge *Spongia officinalis*. Compound **11** exhibited antimicrobial activity against *E*. *coli* and *S*. *aureus* subsp*. aureus* with the MIC values of 16 and 22 μg/mL, respectively [36]. Gallaecimonamides A–C (**12**–**14**) were three new DKPs collected from the marine bacterium *Gallaecimonas mangrovi* HK-28, which was isolated from the mangrove sediment from Haikou, Hainan Province, China. Compound **12** showed significant selectively antimicrobial activity against *V. harveyi* with the MIC value of 50 μM [37]. Three novel chlorine-containing DKPs namely *cis*-cyclo(Pro-3-chloro-Tyr) (**15**), *trans*-cyclo(Pro-3-chloro-Tyr) (**16**) and *cis*-cyclo(3-chloro-Tyr-Ile) (**17**) were isolated from *Bacillus subtilis* BI0980 collected from a depth of 18 m marine sediment between the islands of Kerkyra and Erikoussa. Compounds **15** and **16** showed no inhibitory activity for the tested fungi (*Candida albicans* and *Aspergillus niger*) [38]. All 12 DKPs from bacteria described above are presented in Figure 1.

### 2.3. Actinomycetes

A novel DKP, iso-naseseazine B (**18**), was isolated from the medium of *Streptomyces* sp. SMA-1 from the marine sediment of the Yellow Sea, China. Compound **18** could oppose the fluconazole-resistant *C*. *albicans* and the diameter of the inhibition zone was 9 mm [39]. The other *Streptomyces* sp. USC-636 strain was collected from marine sediment in Sunshine Coast, QLD, Australia. Naseseazine C (**21**) was extracted from the culture of this strain, which exhibited a novel C-6’/C-3 linkage between the two DKP subunits and possessed the activity of antiplasmodial with an IC_50_ value of 3.52 μM. However, the analog naseseazines A (**19**) and B (**20**) inhibited the malaria parasite at a dose of 20 μM. The special linkage between C-6′ and C-3 may be critical to increase bioactivity [40]. (6*R*,3*Z*)-3-benzylidene-6-isobutyl-1-methyl piperazine-2,5-dione (**22**), a new DKP, was produced by the *Streptomyces* sp. strain SCSIO 04496, which was collected from a deep-sea sediment sample of the South China Sea [41]. ΔmarF, the methyltransferase gene of maremycins, knockout in strain *Streptomyces* sp. B9173 (collected from the pacific coast of Chile) obtained the mutant LS26. Six new demethylmaremycins (**23**–**28**) were isolated from the mutant LS26, however, the specific name of these compounds could not be found [42]. *Streptomyces* sp. MNU FJ-36 was collected from the *Katsuwonus* sp. intestinal fabric. Three novel compounds, 3-(3-hydroxy-4-methoxybenzyl)-6-isobutyl-2,5-diketopiperazine (**29**)**,** 3-(1,3-benzodioxol-5-ylmethyl)-6-isobutyl-2,5-diketopiperazine (**30**) and 3-(1,3-benzodioxol-5-ylmethyl)-6-isopropyl-2,5-diketopiperazine (**31**), were isolated from the *Streptomyces* sp. MNU FJ-36. All of these compounds **29**–**31** could inhibit the growth of the A549 cell lines with IC_50_ values of 89.4, 35.4 and 28.4 mg/mL, respectively. Compounds **30** and **31** also exhibited a weak cytotoxicity against the HCT-116 (human colon carcinoma) cell lines with IC_50_ values of 75.4 and 45.4 mg/mL, respectively [43]. A novel DKP glycosidem, maculosin-*O*-*α*-L-rhamnopyranoside (**32**), was discovered from the *Streptomyces* sp. ZZ446, which was collected in coastal soil from Zhoushan Islands, Zhejiang Province. Compound **32** possessed antimicrobial activity against MRSA, *E*. *coli* and *C*. *albicans* with MIC values of 37, 28 and 26 μg/mL, respectively [44,45]. Actinozine A (**33**) was a new DKP, which was isolated from the *Streptomyces* species Call-36 from the Red Sea sponge *Callyspongia* species. Compound **33** exhibited a moderate antimicrobial activity against *S*. *aureus* and *C*. *albicans* with inhibition zones of 23 and 19 mm at 100 μg/disc, respectively, and showed a weak activity for the HCT-116 (IC_50_ = 146 μM) and MCF-7 (breast cancer, IC_50_ = 88.8 μM) cell lines [46]. *Streptomyces* sp. SY1965 was collected from the Mariana Trench sediment-associated at a depth of 11,000 m and two new DKPs, streptodiketopiperazines A (**34**) and B (**35**) were isolated from the strain. The crude extraction of this strain from the Gauze’s liquid medium with sea salt could suppress the human glioma U87MG and U251 cells with an inhibition rate of over 100%. Both compounds **34** and **35** exhibited antifungal activity against *C. albicans* with a MIC value of 42 μg/mL [47].

**Figure 2:**
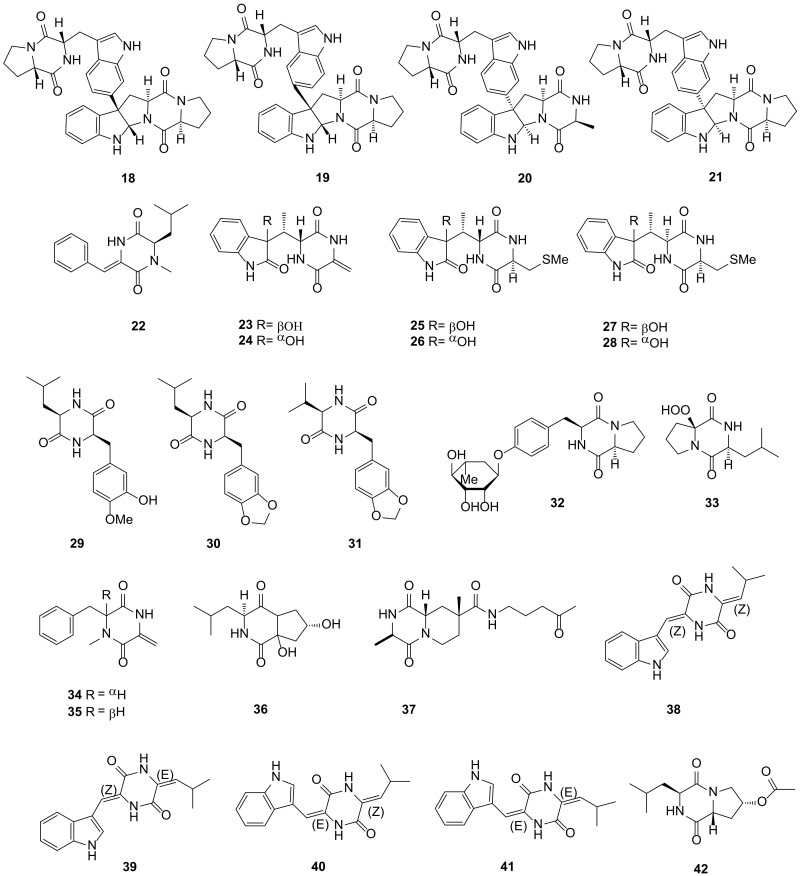
DKP structures from actinomycetes (**18***–***42**).

A novel DKP, cyclo-(4-*trans*-6-dihydroxy-proline-D-leucine) (**36**)**,** was discovered from the *Microbulbifer variabilis* C-03, which isolated from the *Palythoa tuberculosa* in the intertidal zone of Wanlitong [48]. One novel DKP namely nocarazepine A (**37**) was isolated from the *Nocardiopsis alba* collected from the gorgonian *Anthogorgia caerulea,* which was sampled from the coast of Xieyang Island, Guangxi Province [49]. Strain AJS-327 was a rare actinomycete (maybe a new linkage within *Streptomycetaceae*) and collected from the sponge fragment on the beach from La Jolla, CA. It was proved to be a likely novel species because it exhibited an extremely poor 16S rRNA sequence similarity to other members of the actinomycete family *Streptomycetaceae*. Four new DKPs, photopiperazines A–D (**38**–**41**), were isolated from this strain. The olefin geometrical isomers of these four compounds could be interconverted under light conditions, on account of the photopiperazines being sensitive to light. In terms of activity, the mixture of four compounds included compounds **38** (33.5%), **39** (39.7%), **40** (8.4%) and **41** (18.4%). This mixture exhibited a remarkable activity to the cancer cell of U87 (glioblastoma brain cancer), SHOV3 (ovarian cancer), MDA-MB-231(breast cancer) and HCT116 (human colon carcinoma) with IC_50_ values of 1.2 × 10^−4^, 2.2 × 10^−4^, 1.6 and 1.6 μg/mL, respectively [50]. Cyclo-(D-8-acetoxyl-Pro-L-Leu) (**42**) was a novel DKP isolated from *Treptomyces* sp. SCSIO 41400, which was obtained from a mangrove derived-soil from the Fuli Mangrove Bay Wetland Park, Haikou, Hainan Province of China. Compound **42** could anchor in the binding site of the pancreatic lipase (PL) enzyme and then prevent the substrate from entering and inhibit the PL enzyme activity with an IC_50_ value of 27.3 μg/mL [51]. All 25 DKPs from the actinomycetes described above are presented in Figure 2.

### 2.4. Fungi

#### 2.4.1. Fungi from Sediment Origin

Graphiumins A–J (**43**–**52**), ten new sulfur-containing DKPs, were isolated from the fungus *Graphium* sp. OPMF00224 collected from a depth of 17 m marine sediment on Ishigaki Island, Okinawa, Japan. Compounds **45**–**47** and **49**–**52** exhibited no inhibition for MRSA; however, they could inhibit the production of the yellow pigment (virulence factors of MRSA) with a white zone of 10, 14, 10, 12, 11, 24 and 23 mm (50 μg/8 mm) [52,53]. Gu et al. firstly utilized the high-speed countercurrent chromatography (HSCCC) to separate and purify the marine fungus secondary metabolites and then two new sulfur-containing DKPs named cladosporins A (**53**) and B (**54**) were discovered from the marine fungus *Cladosporium* sp. collected from marine sediment in Yangshashan Bay, Ningbo, Zhejiang Province, China. Compounds **53** and **54** showed cytotoxicity activities against HepG2 (hepatocellular carcinoma) cell lines with the IC_50_ values of 21 and 48 μg/mL, respectively. The marine fungus *Alternaria alternate* HK-25 was isolated from mangrove sediment (Sanya, Hainan, China). By using HSCCC, five DKPs named 12,13-dihydroxy-fumitremorgin C (**55**), gliotoxin (**56**), demethoxyfumitremorgin C (**57**), bisdethiobis(methylthio)gliotoxin (**58**) and fumitremorgin C (**59**) were isolated from *A. alternate* HK-25 and first discovered from fungi. Purities of these compounds were all above 94% [54,55].

Haenamindole (**60**) was discovered from the fungus *Penicillium* sp. KCB12F005 isolated from a marine sediment on the coast of Haenam, Korea. Compound **60** exhibited weak inhibitory activity against hepatitis C virus with the IC_50_ value of 76.3 μM [56,57]. Dichotocejpins A–C (**61**–**63**), three novel DKPs, were isolated from the fungus *Dichotomomyces cejpii* FS110 collected from a depth of 3941 m sediment in the South China Sea. Compound **61** exhibited weak cytotoxicity against SF-268 (human glioma), MCF-7 and HepG2 tumor cell lines with IC_50_ values of 35.7, 29.5 and 28.9 μM, respectively, and showed remarkably more α-glucosidase inhibition activity than acarbose (oral antidiabetic agent, IC_50_ = 463 μM) with the IC_50_ value of 138 μM [58]. Cristazine (**64**) was discovered from the fungus *Chaetomium cristatum,* which was isolated from a marine mudflat on Suncheon Bay, Korea. Compound **64** exhibited antioxidant activity against 2,2-diphenyl-1-picrylhydrazyl (DPPH) with the IC_50_ value of 19 μM, which was similar to ascorbic acid (positive control, IC_50_ = 20 μM). It also possessed potent cytotoxicity activities against Hela (cervical carcinoma) and A431 (epidermoid carcinoma) cell lines with the IC_50_ value of 0.5 μM. Compound **64** could trigger the death of the apoptotic cell via the Type I death receptor pathway and inhibit the cell cycle progression by arresting the G_1_/S phase and upregulating the inhibitory proteins of cyclin-dependent kinases. By these two ways, compound **64** could inhibit the growth of A431 cells [59,60].

Nineteen novel DKPs namely eutypellazines A–S (**65**–**83**) were isolated from fungus *Eutypella* sp. MCCC 3A00281, which was collected from a depth of 5610 m of marine sediment from the South Atlantic Ocean. Compounds **65**–**76** could inhibit the replication of human immunodeficiency virus type 1 (HIV-1) with the IC_50_ values ranging 3.2–18.2 μM. A sulfide bridge existing in compounds **65**–**67** and **71**–**72** (IC_50_ = 10.7–18.2 μM) might be the reason for exhibiting lower inhibitory activities than compounds **68**–**69** and **73**–**76** (IC_50_ = 3.2–8.7 μM). Compound **74** exhibited the ability to reactivate the latent HIV-1 transcription, which was rarely discovered from nature products. These compounds may become new anti-HIV candidates via modifying the original new scaffolds [61,62]. Fusaperazine F (**84**) was isolated from the fungus *Penicillium crustosum* HDN153086 collected from the Prydz Bay sediment of the Antarctic and showed cytotoxicity activity against K562 cell lines (human chronic myelogenous leukemia cells, IC_50_ = 12.7 μM) [63]. Roquefortine J (**85**) was another new DKP discovered from the fungus *Penicillium granulatum* MCCC 3A00475 isolated from a -2284 m deep-sea sediment from the Prydz Bay of Antarctica and exhibited moderate cytotoxicity activity against HepG2 cell lines with an IC_50_ value of 19.5 μM [64,65]. Three pairs of new DKPs namely (±)-7,8-epoxy-brevianamide Q (**86–87**), (±)-8-hydroxy-brevianamide R (**88–89**) and (±)-8-epihydroxy-brevianamide R (**90–91**) were isolated from the fungus *Aspergillus versicolor* MF180151, which was collected from a sediment in Bohai Sea, China. However, these compounds showed no antimicrobial activities [66].

Raistrickindole A (**92**)**,** a novel DKP containing indole tetraheterocyclic ring system, was discovered from the fungus *Penicillium raistrickii* IMB17-034 obtained from a mangrove swamp sediment in Sanya, Hainan Province, China. Compound **92** exhibited the inhibitory activity against hepatitis C virus (HCV) with the EC_50_ value of 5.7 μM [67]. 5′-hydroxy-6′-ene-epicoccin G (**93**), 7-methoxy-7′-hydroxyepicoccin G (**94**), 8′-acetoxyepicoccin D (**95**), 7′-demethoxyrostratin C (**96**) and (±)-5-hydroxydiphenylalazines A (**97–98**) were five new DKPs and isolated from the fungus *Epicoccum nigrum* SD-388, which was collected from a depth of 4500 m of sediment in the West Pacific. Compound **96** possessed the excellent cytotoxicity activity against Huh7.5 (liver tumor) cell lines with an IC_50_ value of 9.52 μM, however, it also inhibited the growth of human normal liver LO2 cell lines. The disulfide bridge in the main structure may be crucial for its activity [68]. (±) Eurotinoids A (**99**–**100**), (±) eurotinoids B (**101**–**102**) and (±) eurotinoids C (**103**–**104**), three pairs of novel DKPs spirocyclic alkaloid enantiomers, were isolated from the fungus *Eurotium* sp. SCSIO F452 obtained from the sediment sample in South China Sea. These compounds showed excellent antioxidant activities against DPPH with the IC_50_ values between 5.8 and 24.9 μM [69].

Seven novel DKPs, versicamides A−G (**105**−**111**), were isolated from the fungus *Aspergillus versicolor* HDN08-60 collected from the marine sediment in the South China Sea. Versicamide H (**112**) was obtained from the methylation reaction of **111**. Only compound **112** showed modest cytotoxicity activities against the HL-60, HCT-116, Hela and K562 cell lines with IC_50_ values of 8.7, 17.7, 19.4 and 22.4 μM, respectively, and compound **112** exhibited selective PTK inhibitory activity with the highest inhibitory rate of 60% at a concentration of 10 μM. Compound **112** had stronger activities than compounds **105**–**111**, which might be attributed to an unprecedented skeleton featuring a 2,5-dihydro-1H-azepino-[4,3-b]quinoline system [70]. Fifteen novel DKPs namely rubrumlines A–O (**113**–**127**) were isolated from the fungus *Eurotium rubrum,* which was collected from a depth of 2067 m marine sediment in the South Atlantic Ocean. Compound **116** possessed antivirus activity against the influenza A/WSN/33 virus with the inhibitory rate of 52.64% [71]. Ten new DKPs, 12*β*-hydroxy-13*α*-ethoxyverruculogen TR-2 (**128**), 12*β*-hydroxy-13*α*-butoxyethoxyverruculogen TR-2 (**129**), hydrocycloprostatin A (**130**), hydrocycloprostatin B (**131**), 25-hydroxyfumitremorgin B (**132**), 12*β*-hydroxy-13*α*-butoxyethoxyfumitremorgin B (**133**), 12*β*-hydroxy-13*α*-methoxyverruculogen (**134**), 26*α*-hydroxyfumitremorgin A (**135**), 25-hydroxyfumitremorgin A (**136**) and diprostatin A (**137**) were isolated from the coculture of the fungus *Penicillium* sp. DT-F29 with the bacteria *Bacillus* sp. B31, which were collected from the marine sediments of Dongtou County and Changzhi Island, respectively. In a dose of 20 μM, compounds **133** and **137** showed remarkable BRD4 protein inhibitory activities [72].

**Figure 3:**
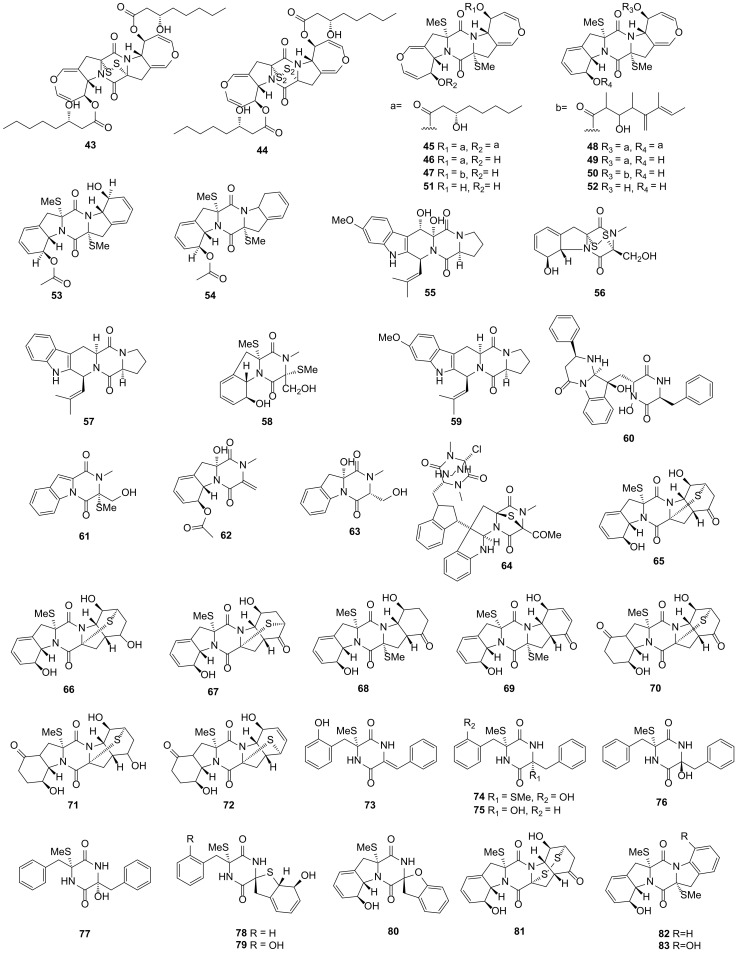
DKP structures from sediment-derived fungi (**43***–***83**).

**Figure 4:**
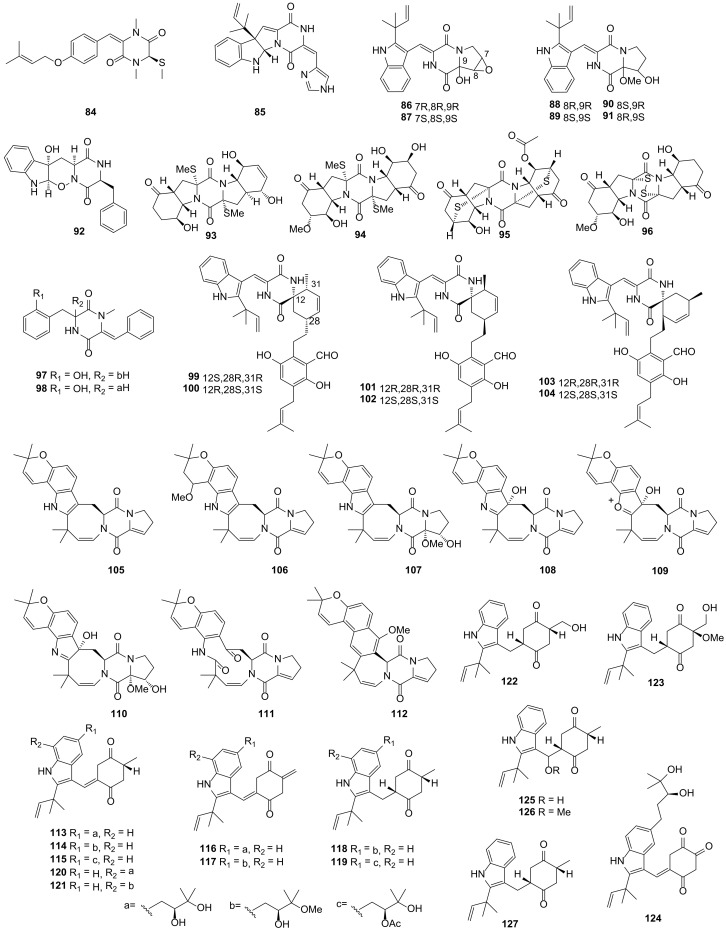
DKP structures from sediment-derived fungi (**84***–***127**).

**Figure 5:**
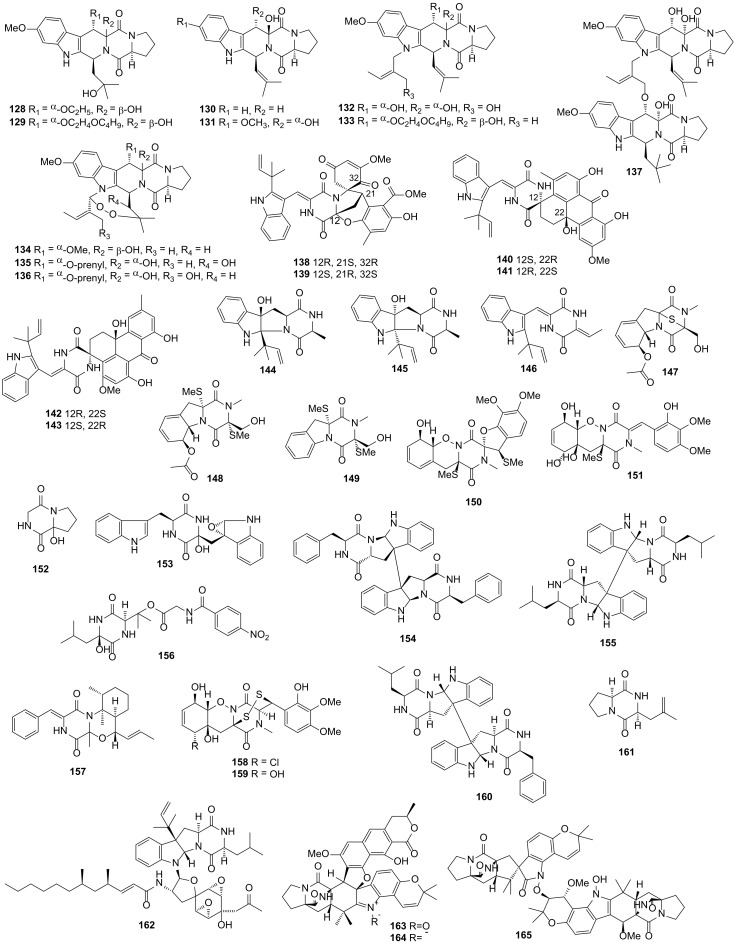
DKP structures from sediment-derived (**128***–***145**), sponge-derived (**146***–***161**) and beach-derived fungi (**162***–***165**).

Three novel pairs of spirocyclic DKPs namely (±) variecolortins A−C (**138**−**143**) were obtained from the fungus *Eurotium* sp. SCSIO F452, which was isolated from marine sediment in the South China Sea. Compound **138** had a 2-oxa-7-azabicyclo[3.2.1]octane core, which was an unprecedented highly functionalized seco-anthronopyranoid carbon skeleton. In addition, compounds **139** and **140** had a rare 6/6/6/6 tetracyclic cyclohexene−anthrone carbon scaffold. Compound **138** showed remarkable antioxidant activity against DPPH with an IC_50_ value of 58.4 μM. Compound **140** and **142** exhibited modest cytotoxicity activities against SF-268 (IC_50_ = 12.5 and 15.0 μM) and HepG2 (IC_50_ = 30.1 and 37.3 μM) cell lines. The preliminary molecular docking study revealed that the potential antioxidative target of compounds **138** and **139** might be peroxiredoxin and the potential cytotoxic target of compounds **140** and **142** might be farnesyltransferase [73]. Eurotiumins A–C (**144**–**146**), three new DKP alkaloids, were also isolated from the fungus *Eurotium* sp. SCSIO F452. Compounds **144**–**146** showed antioxidant activities against DPPH with IC_50_ values of 37, 69 and 13 μM, respectively. Based on these results, the absolute configurations of the C-2 and C-3 of compounds **144** and **145** might have an influence on antioxidant activities [74]. All 104 DKPs from sediment-derived fungi described above are presented in Figure 3, Figure 4 and Figure 5.

#### 2.4.2. Fungi from Sponge Origin

A novel DKP namely 6-acetylmonodethiogliotoxin (**147**) was obtained from the fungus *Dichotomomyces cejpii,* which was collected from the marine sponge *Callyspongia* cf. *C*. *flammea* at Bare Island, Sydney, Australia. Two DKPs previously only known as semisynthetic compounds, 6-acetylbisdethiobis(methylthio)gliotoxin (**148**) and 5a,6-anhydrobisdethiobis(methylthio)gliotoxin (**149**), were found in nature for the first time. In human chronic myeloid leukemia cells, compounds **147** and **148** showed downregulated TNFα-induced NF-κB activity with the IC_50_ values of 38.5 and 65.7 μM, respectively [75]. Peniciadametizine A (**150**) and B (**151**) were isolated from the fungus *Penicillium adametzioides* AS-53 collected from an unidentified sponge in Hainan Island of China. Compound **150** had a unique spiro[furan-2,7’-pyrazino[1,2-b][1,2]oxazine] skeleton, which was found from a natural source for the first time and compound **151** was the highly oxygenated analogue of **150**. In a dose of 100 μg/mL, compounds **150** and **151** could kill *Artemia salina* with the lethal ratio of 45.5 and 62.4%, respectively. These two compounds also showed antifungal activity against *Alternaria brassicae* with MIC values of 4.0 and 32.0 μg/mL, respectively [76]. Cyclo-(2-hydroxy-Pro-Gly) (**152**)**,** a novel minor DKP, was obtained from the fungus *Simplicillium* sp. YZ-11, which was collected from an intertidal sponge *Hymeniacidon perleve* from Dalian, Liaoning Province, China [77]. *Neosartorya glabra* KUFA 0702 was isolated from the sponge *Mycale* sp. collected from 15 to 20 m coral reef at Samaesarn Island and a new DKP, fellutanine A (**153**), was obtained. Compound **153** showed no antibacterial (MIC > 256 μg/mL) or antifungal (MIC > 512 μg/mL) activity against the tested microbe including *E*. *coli* ATCC 25922, *Staphylococcus aureus* ATCC 25923, *A*. *fumigatus* ATCC 46645, *Trichophyton rubrum* ATCC FF5 and *C*. *albicans* ATCC 10231 [78]. Asperflocin (**154**), a novel asymmetric DKP dimer, was isolated from the fungus *Aspergillus versicolor* 16F-11, which was obtained from the sponge *Phakellia fusca* from Yongxing Island in the South China Sea. Compound **154** could inhibit the A375 (human melanoma) cell lines growth with the IC_50_ value of 10.29 ± 2.37 μM [79].

One novel DKP dimer (**155**) possessing the same planar structure and different stereochemistry with an unnamed and ambiguous compound was isolated from the fungus *Aspergillus violaceofuscus,* which was collected from sponge *Reniochalina* sp. from Xisha Islands in the South China Sea. In a dose of 10 μM, compound **155** exhibited anti-inflammatory activity via decreasing LPS-induced expression of IL-10 in THP-1 cells with inhibitory rates of 78.1% [80]. Waspergillamide B (**156**), a new DKP containing an unusual *p*-nitrobenzoic acid structure, was obtained from the fungus *Aspergillus ochraceus* collected from the sponge *Agelas oroides* from the Mediterranean. Compound **156** had no cytotoxic activity against A2780 (human ovarian carcinoma) cell lines [81]. One new DKP alkaloid namely penicillivinacine (**157**) was obtained from the fungus *Penicillium vinaceum* that was isolated from the marine sponge *Hyrtios erectus* from Yanbu. Compound **157** exhibited significant cytotoxicity activity against MDA-MB-231 cell lines with an IC_50_ value of 18.4 μM [82]. Adametizines A (**158**) and B (**159**), two novel sulfur-containing DKPs, were separated from the fungus *Penicillium adametzioides* AS-53, which was isolated from an unidentified marine sponge collected from Hainan Island of China. Compound **158** could inhibit the growth of brine shrimp (*A*. *salina*) with the LD_50_ (lethal dose 50%) value of 4.8 μM. In addition, compound **158** showed antimicrobial activities against *Staphylococcus aureus*, *Aeromonas hydrophilia* and *V*. *parahaemolyticus* with an MIC value of 8 μg/mL and compound **159** showed antimicrobial activities against *S*. *aureus* with an MIC value of 64 μg/mL. These results revealed that the Cl substitution at C-7 remarkably increased the brine shrimp lethality and antimicrobial activity [83]. SF5280-415 (**160**) was a novel DKP dimer and isolated from the fungus *Aspergillus* sp. SF-5280, which was obtained from an unidentified sponge at Cheju Island, Korea. Compound **160** showed enzyme inhibitory activity against the PTP1B enzyme with an IC_50_ value of 14.2 μM [84]. All 14 DKPs from sponge-derived fungi described above are presented in Figure 5.

#### 2.4.3. Fungi from Beach Origin

A novel DKP namely penicimutide (**161**) was obtained from a neomycin-resistant mutant fungus *Penicillium purpurogenum* G59, which was isolated from a soil sample from the tideland of Bohai Bay. In a dose of 100 μg/mL, compound **161** exhibited excellent cytotoxicity activity against HeLa cell lines with an inhibition rate of 39.4% [85]. Penicimutanin C (**162**), a new DKP contained alkaloidal, was isolated from the neomycin-resistant mutant strain 3-f-31 fungus *P. purpurogenum* G59. Compound **162** exhibited cytotoxic activities against HeLa, BGC-823 (gastric adenocarcinoma), MCF-7, K562 and HL-60 (acute promyelocytic leukemia) cell lines with IC_50_ values of 11.9, 5.0, 8.6, 8.7 and 6.0 μM, respectively. In a dose of 100 μg/mL, compound **162** showed cytotoxicity activities against these five cell lines with inhibition rates of 88.1%, 83.9%, 80.5%, 87.7% and 87.3%, respectively [86].

Waikikiamides A−C (**163**–**165**) were obtained from the fungus *Aspergillus* sp. FM242 collected from a sample at Waikiki beach in Oahu, Honolulu, Hawaii. Compounds **163** and **164** contained a new skeleton with a hendecacyclic ring system and compound **165** was composed of two notoamide analogs with an N-O-C bridge to feature the unique heterodimer. Compounds **163** possessed antiproliferative activities against HT1080 (fibrosarcoma), PC3 (prostatic tumor), Jurkat (immortalized T lymphocyte) and A2780S cell lines with IC_50_ values of 0.519, 1.855, 0.62 and 0.78 μM, respectively. Compound **165** exhibited antiproliferative activities against these four cell lines with IC_50_ values of 1.135, 1.805, 1.79 and 1.127 μM, respectively. Compound **164** showed no activities to these four cell lines. The reason for the difference in activity was that compounds **163** and **165** possessed an N–O bond but compound **164** did not [87]. All 5 DKPs from fungi of beach origin described above are presented in Figure 5.

#### 2.4.4. Fungi from Mangrove Origin

A new DKP namely 5*S*-hydroxynorvaline-*S*-Ile (**166**), together with two firstly discovered in nature namely 3*S*-hydroxylcyclo(*S*-Pro-*S*-Phe) (**167**) and cyclo(*S*-Phe-*S*-Gln) (**168**), were obtained from mangrove endophytic fungus *Penicillium* sp. GD6, which was isolated from the stem bark of *Bruguiera gymnorrhiza* collected from Zhanjiang, China. These compounds showed no activity against the tested MRSA [88]. (−)-asperginulin A (**169**) and (+)-asperginulin A (**170**), two dimers DKPs that contained enantiomeric indole, were isolated from the mangrove endophytic fungus *Aspergillus* sp. SK-28, which was obtained from *Kandelia candel* from the Shankou Mangrove Nature Reserve in Guangxi Province, China. Compound **170** could inhibit the growth of the barnacle *Balanus reticulatus* with antifouling activity and low toxicity [89]. Three novel DKPs, saroclazines A–C (**171–173**), were isolated from the fungus *Sarocladium kiliense* HDN11-84, which was obtained from a root soil sample of mangrove *Thespesia populnea* from Guangxi Province, China. Compound **171** exhibited cytotoxicity activity against Hela cell lines with the IC_50_ value of 4.2 μM [90].

**Figure 6:**
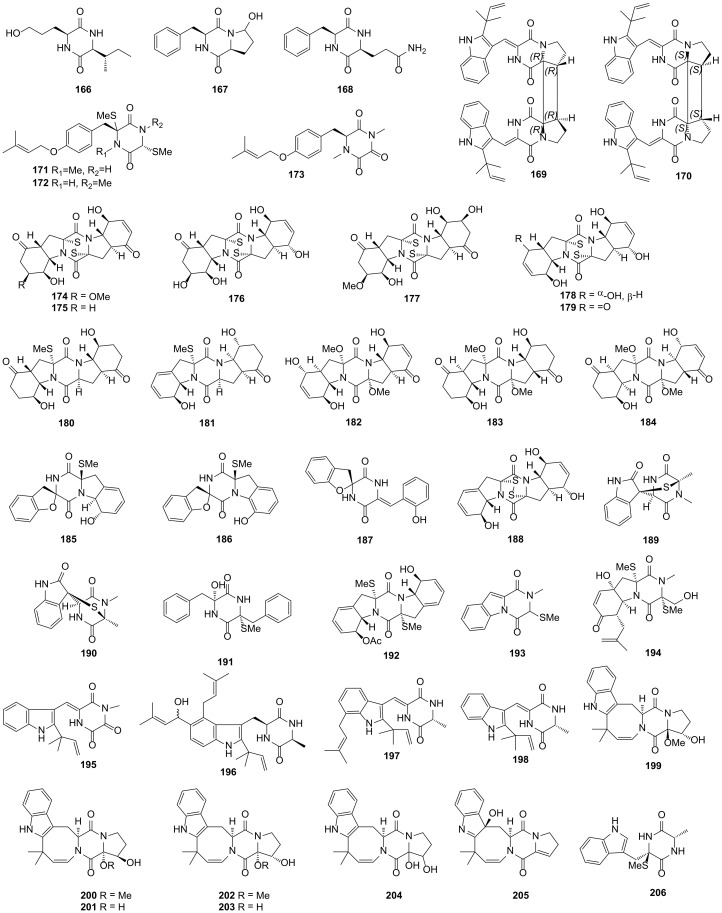
DKP structures from mangrove-derived (**166***–***188**) and coral-derived fungi (**189***–***206**).

Fifteen novel DKPs, brocazines A−F (**174**−**179**), penicibrocazines A–E (**180**–**184**), spirobrocazines A−C (**185**−**187**) and brocazine G (**188**) were obtained from the fungus *Penicillium brocae* MA-231, which was isolated from the marine mangrove plant *Avicennia marina* in Hainan Island. Compounds **174–177** showed potent cytotoxic activities against Du145 (human prostate carcinoma), Hela, HepG2, MCF-7, NCI-H460 (human non-small cell lung cancer), SGC-7901 (human gastric carcinoma), SW1990 (human pancreatic adenocarcinoma) and U251 cell lines with the IC_50_ values in the range of 0.89–12.4 μM [91]. Compounds **182** and **184** exhibited antimicrobial activities against *S*. *aureus* and *Gaeumannomyces graminis* with the MIC values ranging from 0.25 to 32.0 μg/mL, respectively, and compound **183** also showed antimicrobial activities against *S*. *aureus* and *Micrococcus luteus* with the MIC values of 0.25 μg/mL [92]. In addition, compounds **185** and **187** showed modest antimicrobial activities against *E*. *coli* and *Vibrio harveyi* with the MIC values in the range of 32–64 μg/mL. Compound **188** exhibited significant cytotoxic activities against A2780 and A2780 CisR cell lines with the IC_50_ values of 664 and 661 nM, respectively, and had potent antimicrobial activity against *S*. *aureus* with an MIC value of 0.25 μg/mL [91,93]. These results showed that compounds possessing two double bonds at C-6 and C-6′ or one double bond at C-6/6′ conjugating with a keto group at C-5/5′ might exhibit higher cytotoxic or antimicrobial activity. All 23 DKPs from mangrove-derived fungi described above are presented in Figure 6.

#### 2.4.5. Fungi from Coral Origin

Pseudellones A (**189**) and B (**190**), two new DKPs containing an irregular bridge, were separated from the fungus *Pseudallescheria ellipsoidea* F42−3 derived from the soft coral *Lobophytum crassum* in Hainan Sanya National Coral Reef Reserve, China [94]. Pseuboydones C (**191**) and D (**192**) were isolated from the fungus *Pseudallescheria boydii* F19-1 collected from the soft coral *L*. *crassum*. Compound **191** exhibited remarkable cytotoxic activity against Sf9 cell lines with an IC_50_ value of 0.7 μM [95]. Fungus *Dichotomomyces cejpii* F31-1 was collected from the soft coral *L*. *crassum* and two novel DKPs namely dichocerazines A (**193**) and B (**194**) were then obtained from the GPY medium (added L-tryptophan and L-phenylalanine) of the fungus. These two compounds showed no activity for the tested HCT116, RD (human rhabdomyosarcoma), ACHN (human renal carcinoma) and A2780T cell lines [96]. Three new DKPs alkaloids namely 11-methylneoechinulin E (**195**), variecolorin M (**196**) and (+)-variecolorin G (**197**) and a DKP first discovered in nature namely (+)-neoechinulin A (**198**) were obtained from the fungus *Aspergillus* sp. EGF 15-0-3, which was isolated from a soft coral in the South China Sea. Compounds **195–198** showed cytotoxic activity against NCI-H1975 gefitinib resistance cell lines at the concentration of 50 μM [97].

16*α*-hydroxy-17*β*-methoxy-deoxydihydroisoaustamide (**199**), 16*β*-hydroxy-17*α*-methoxy-deoxydihydroisoaustamide (**200**), 16*β*,17*α* -dihydroxy-deoxydihydroisoaustamide (**201**), 16*α* -hydroxy-17*α*-methoxy-deoxydihydroisoaustamide (**202**), 16*α*,17*α* -dihydroxy-deoxydihydroisoaustamide (**203**), 16,17-dihydroxy-deoxydihydroisoaustamide (**204**) and 3*β*-hydroxy-deoxyisoaustamide (**205**)**,** seven new DKPs containing a prenylated indole ring, were obtained from the fungus *Penicillium dimorphosporum* KMM 4689, which was separated from an unidentified soft coral in the South China Sea. When the murine neuroblastoma Neuro-2a cells were treated with the mixture of 500 μM paraquat (PQ) and 1 μM each of compounds **202**, **203** and **204**, the cell viability was increased by 38.6%, 30.3% and 36.5%, respectively, compared with the treatment of PQ alone. The hydroxy groups at C-16 and C-17 played a key role in neuroprotective activity by the analysis of structure–activity relationships [98]. Pseudellone D (**206**), a novel DKP alkaloid possessing a rare monomethylthio group, was isolated from the fungus *Pseudallescheria ellipsoidea* F42-3 sourced from the soft coral *Lobophytum crissum* [99]. All 18 DKPs from coral-derived fungi described above are presented in Figure 6.

#### 2.4.6. Fungi from Alga Origin

Dehydroxymethylbis(dethio)bis(methylthio)gliotoxin (**207**) and (3S,6R)-6-(para-hydroxybenzyl)-1,4-dimethyl-3,6-bis(methylthio)piperazine-2,5-dione (**208**), two novel sulphurated DKPs, were obtained from the fungus *Trichoderma virens* Y13-3 derived from the marine red alga *Gracilaria vermiculophylla* in Yangma Island [100]. Methylcordysinin A (**209**) was separated from *Trichoderma asperellum* cf44-2, which was collected from brown alga *Sargassum* sp. in Zhoushan Islands [101]. Four novel DKP alkaloids named citriperazines A-D (**210–213**) were separated from *Penicillium* sp. KMM 4672 obtained from the Vietnamese marine brown algae *Padina* sp. [102]. Compounds **207**–**213** showed no inhibitory activities for the tested bacteria or cancer cell lines. The endophytic fungus *Acrostalagmus luteoalbus* TK-43 was isolated from the green algal *Codium fragile* collected in Sinop, Turkey, and six novel N-methoxy-containing indole DKPs, namely (±) acrozines A–C (**214**–**219**), were obtained from the strain. Compound **216** exhibited moderate antimicrobial activity against the plant pathogen *Fusarium solani* (MIC = 32 μg/mL). Compounds **214** and **215** possessed antiacetylcholinesterase activities with the IC_50_ values of 2.3 and 13.8 μM, respectively. This result indicated that the bioactivity was concerned with the absolute configurations of these compounds [103].

**Figure 7:**
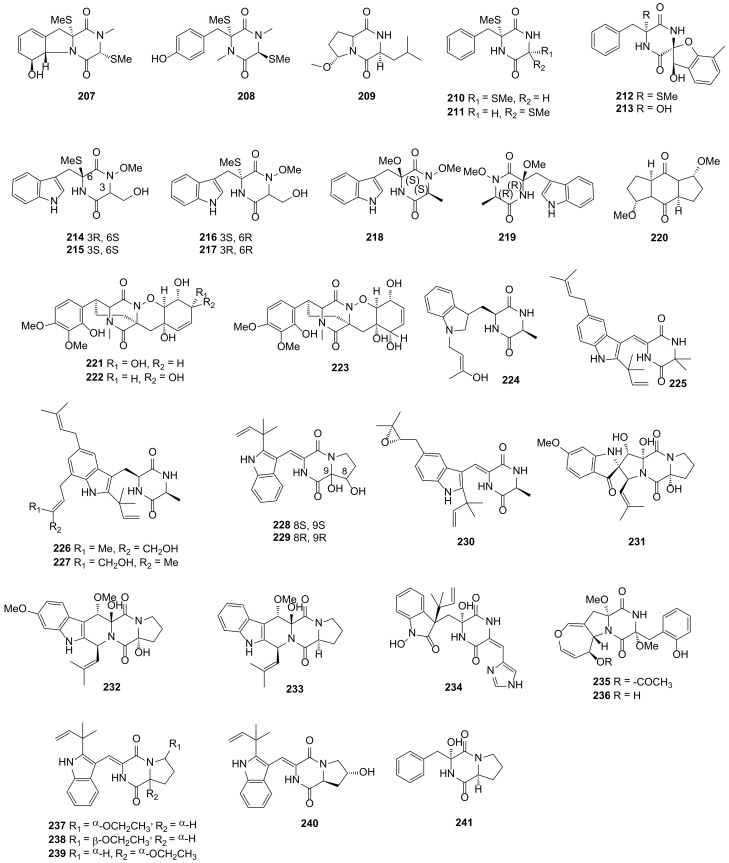
DKP structures from alga-derived (**207***–***229**) and other sourced fungi (**230***–***241**).

One new DKP namely cyclo(L-5-MeO-Pro-L-5-MeO-Pro) (**220**) was isolated from the fungus *Trichoderma asperellum* A-YMD-9-2, which was obtained from marine macroalga *Gracilaria verrucose* collected from Yangma Island. Compound **220** exhibited inhibitory activities against *Chattonella marina*, *Heterosigma akashiwo*, *Karlodinium veneficum* and *Prorocentrum donghaiense* with the EC_50_ values of 47.3, 276, 327 and 351 μM, respectively [104]. Three novel sulfur-containing DKPs namely pretrichodermamides D–F (**221–223**) were separated from the fungus *Penicillium* sp. KMM 4672, which was obtained from the Vietnamese brown alga *Padina* sp. These compounds did not show potent activities for the human prostate cancer 22Rv1 cells [105]. Four new DKP alkaloids namely N-(4′-hydroxyprenyl)-cyclo(alanyltryptophyl) (**224**), isovariecolorin I (**225**), 30-hydroxyechinulin (**226**) and 29-hydroxyechinulin (**227**) were obtained from the fungus *Eurotium cristatum* EN-220, which was collected from marine alga *Sargassum thunbergia* on the coast of Qingdao, China. Compound **225** showed brine shrimp (*A*. *salina*) lethal activity with the LD_50_ value of 19.4 μg/mL and had moderate antioxidative activities with an IC_50_ value of 20.6 μg/mL [106]. (±)-Brevianamides X (**228** and **229**) were obtained from the fungus *Aspergillus versicolor* OUCMDZ-2738 isolated from alga *Enteromorpha prolifera,* which was collected from Shilaoren beach, Qingdao, China, and showed no antimicrobial activities [107]. All 23 DKPs from alga-derived fungi described above are presented in Figure 7.

#### 2.4.7. Fungi from Other Origin

One novel DKP namely isoechinulin D (**230**) was isolated from the marine fungus *Eurotium rubrum* MPUC136, which was collected from the seaweed in Chosei-mura, Chosei-gun, Chiba Prefecture, Japan, and showed weak inhibitory activity against melanin synthesis with an IC_50_ value of 60 μM. [108]. Spirotryprostatin G (**231**), cyclotryprostatins F (**232**) and G (**233**), three new DKPs alkaloids, were isolated from the fungus *Penicillium brasilianum* HBU-136 separated from the Bohai Sea. Compound **231** showed excellent cytotoxic activity against HL-60 cell lines with an IC_50_ value of 6.0 μM and compounds **232** and **233** exhibited remarkable cytotoxic activity against MCF-7 cell lines with IC_50_ values of 7.6 and 10.8 μM, respectively [109]. A new DKP alkaloid namely penilline C (**234**) was obtained from the fungus *Penicillium chrysogenum* SCSIO 07007, which was isolated from a deep-sea hydrothermal vent environment sample of Western Atlantic [110]. Emestrins L (**235**) and M (**236**), two novel DKPs, were separated from the fungus *Aspergillus terreus* RA2905, which was obtained from the sea hare *Aplysia pulmonica* from the Weizhou coral reefs in the South China Sea. Compound **236** showed antifungal activity against *P*. *aeruginosa* ATCC 27853 with the MIC value of 64 μg/mL [111].

Aspamides A–D (**237**–**240**), four novel DKPs alkaloids, were separated from the endophyte fungus *Aspergillus versicolor* DY180635, which was isolated from the sea crab *Chiromantes haematocheir* from the intertidal zone of Zhoushan, Zhejiang, China. For the virtual screening on the 3CL hydrolase (Mpro) of SARS-CoV-2 (potential drug target to fight COVID-19), the docking scores of compounds **237** and **238** were −5.389 and −4.772, respectively, and the score of positive control ritonavir was −7.039. In the future, these two compounds may be helpful in fighting COVID-19 [112]. One novel DKP namely penicillatide B (**241**) was isolated from the fungus *Penicillium* sp., which was collected from the Red Sea tunicate *Didemnum* sp. Compound **241** showed moderate cytotoxic activity against the HCT-116 cell lines with an IC_50_ value of 23.0 μM and exhibited modest antimicrobial activities against *S*. *aureus* and *V*. *anguillarum* with inhibition zones of 19 and 20 mm, respectively [113]. All 12 DKPs from fungi sourced from other origins described above are presented in Figure 7.

## 3. Chemical Structures of Diketopiperazine Derivatives from Marine Organisms

Diketopiperazine derivatives are further modified on the basis of the six-membered piperazine rigid backbone. The following 54 DKP derivatives were described in this paper, of which 53 lacked a carbonyl group and 1 lacked two carbonyl groups in the skeleton (Table 2).

### 3.1. Actinomycetes

A mixture of two new tautomers DKP derivatives named isomethoxyneihumicin (**242** and **243**) were obtained from the actinomycete *Nocardiopsis alba* KM6-1, which was collected from marine sediment in Chichijima, Ogasawara, Japan. The mixture showed excellent cytotoxic activity against Jurkat cell lines with the IC_50_ value of 6.98 μM and in a dose of 15 μM, compounds **242** and **243** made the cell cycle of Jurkat cell lines staying in the G2/M phase with the inhibition ratio of 66% in 12 h. These consequences indicated that the mixed compounds inhibited the growth of Jurkat cell lines via arresting the cell cycle at the G2/M phase [114]. Two novel DKP derivatives namely nocazines A (**244**) and B (**245**) were isolated from *Nocardiopsis dassonvillei* HR10-5, which was obtained from marine sediment in the estuary of Yellow River, Dongying, China. However, compounds **244** and **245** did not exhibit cytotoxic or antimicrobial activities for the tested cancer cell lines and microorganisms [115].

**Figure 8:**
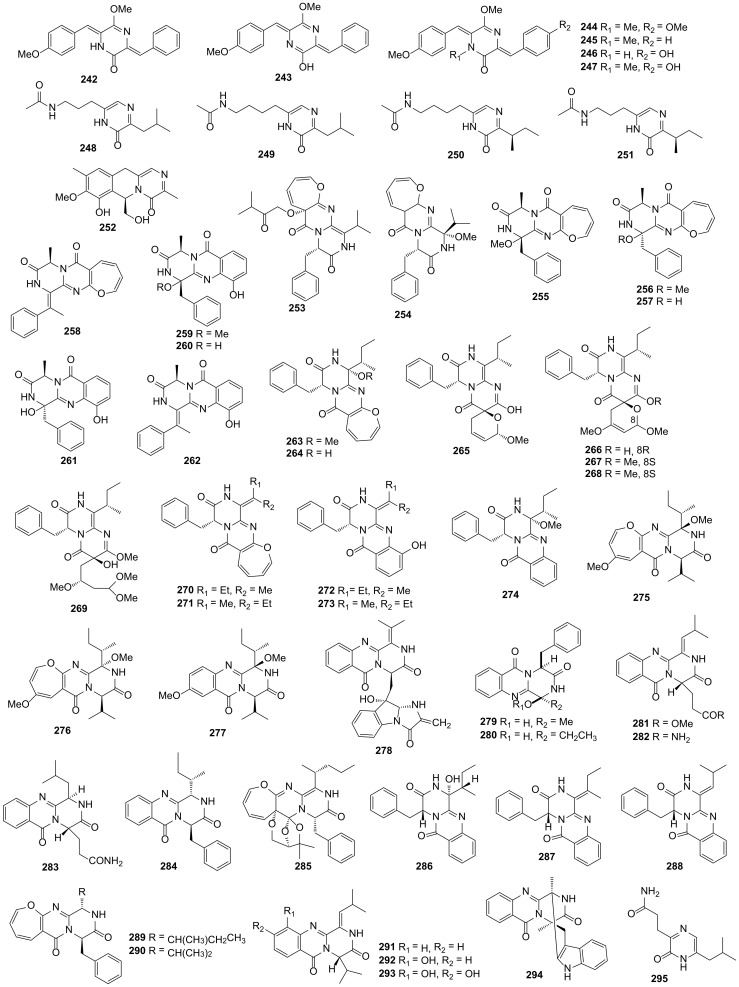
DKP derivative structures from actinomycetes (**242***–***252**) and fungi (**253***–***295**).

Nocazines F (**246**) and G (**247**), two novel DKP derivatives, were isolated from the *Nocardiopsis* sp. YIM M13066, which was collected from the deep-sea sediment. Compound **246** showed remarkable cytotoxic activities against the human cancer cell lines H1299 (non-small cell lung cancer), Hela, HL7702, MCF-7, PC3 and U251 with IC_50_ values of 3.87, 4.77, 7.10, 3.86 and 8.17 μM, respectively. Compound **247** also showed excellent cytotoxic activities against the human cancer cell lines H1299, Hela, HL7702 (human derived liver), MCF-7, PC3 and U251 with the IC_50_ values of 2.60, 3.97, 8.73, 6.67 and 16.7 μM, respectively, and exhibited modest antimicrobial activity against *B*. *subtilis* ATCC 6051 with the MIC value of 25.8 μM [116]. Streptopyrazinones A–D (**248**–**251**) were four new DKP derivatives and isolated from *Streptomyces* sp. ZZ446, which was collected from a coastal soil sample from Zhoushan Islands. Compounds **248**–**251** exhibited moderate antimicrobial activities against *C*. *albicans* and MRSA with the MIC values of 35–45 and 58–65 μg/mL, respectively [44]. One novel tricyclic DKP derivative namely strepyrazinone (**252**) was obtained from *Streptomyces* sp. B223, which was isolated from the marine sediment of Laizhou Bay. Compound **252** displayed remarkable cytotoxic activity against the HCT-116 cell lines with the IC_50_ value of 0.34 μM [117]. All 11 DKP derivatives from actinomycetes described above are presented in Figure 8.

### 3.2. Fungi

Varioloids A (**253**) and B (**254**) were two novel oxepine-containing DKP derivatives and obtained from the endophytic fungus *Paecilomyces variotii* EN-291, which was collected from red alga *Grateloupia turuturu* on the coast of Qingdao. Compounds **253** and **254** showed significant antimicrobial activities against *F*. *graminearum* with the MIC values of 8 and 4 μg/mL, respectively. In addition, compounds **253** and **254** also inhibited the growth of *A*. *hydrophila*, *E*. *coli*, *M*. *luteus*, *S*. *aureus*, *V*. *anguillarum*, *V. harveyi* and *V. parahaemolyticus* with the MIC values in the range of 16–64 μg/mL [118]. Four novel oxepine-containing DKP derivatives namely oxepinamides H−K (**255**–**258**) and four novel 4-quinazolinone DKP derivatives namely puniceloids A−D (**259**–**262**) were isolated from the fungus *Aspergillus puniceus* SCSIO z021, which was collected from deep-sea sediment in Okinawa Trough. Compounds **255**–**257** and **259**–**262** exhibited remarkable transcriptional activation of liver X receptor *α* with the EC_50_ values of 1.7–16 μM. This result suggested that the transcriptional activation activity of these compounds would be decreased when the benzene ring was converted into an oxepin unit. In addition, compound **262** possessed enzyme inhibitory activities against seven enzymes including TCPTP, SHP1, MEG2, SHP2, PTP1B, IDO1 and LDHA with the IC_50_ values in the range of 14–87 μM [119].

Protuboxepins C (**263**) and D (**264**), two novel oxepin-containing DKP derivatives, were obtained from the fungus *Aspergillus* sp. SCSIO XWS02F40, which was isolated from the sponge *Callyspongia* sp. from the sea area near Xuwen County, Guangdong Province, China. Compounds **263** and **264** showed moderate cytotoxic activities against Hela cell lines with the IC_50_ values of 61 and 114 μM, respectively [120,121]. Pyranamides A−D (**265**–**268**), secopyranamide C (**269**) and protuboxepins F−J (**270**–**274**), ten novel DKP derivatives, were isolated from the marine sponge-derived fungus *Aspergillus versicolor* SCSIO 41016, which was also separated from *Callyspongia* sp. Compound **270** showed modest cytotoxic activities against the ACHN, OS-RC-2 and 786-O cell lines (three renal carcinoma cell lines) with the IC_50_ values of 27, 34.9 and 47.1 μM, respectively [122]. Chrysopiperazines A–C (**275**–**277**) were three new DKP derivatives and obtained from the fungus *Penicillium chrysogenum,* which was collected from gorgonian *Dichotella gemmacea* in South China Sea. The oxepine-containing DKPs were found from the genus *Penicillium* for the first time [123]. One novel DKP derivative namely quinadoline D (**278**) was isolated from the fungus *Penicillium* sp. L129, which was collected from the rhizosphere-soil of *Limonium sinense* (Girald) Kuntze from Yangkou Beach, Qingdao, China [124]. Two novel DKP derivatives namely aspamides F (**279**) and G (**280**) were isolated from the endophyte fungus *A. versicolor* DY180635. For the virtual screening on the 3CL hydrolase of SARS-CoV-2, the docking scores of compounds **279** and **280** were −5.146 and −4.962, respectively [112].

Polonimides A–C (**281**–**283**), three novel quinazoline-containing DKP derivatives, were isolated from the fungus *Penicillium polonicum* obtained from the Bohai Sea. Compounds **281**–**283** showed potent chitinase inhibitory activity against GH18 chitinase *Of*Chi-h with the inhibition rates of 91.9%, 79.1% and 86.1%, respectively [125]. Protuboxepin K (**284**) was obtained from the fungus *Aspergillus* sp. BFM-0085, which was collected from a marine sediment sample of Tokyo Bay. In mutant bone morphogenetic protein (BMP) receptor-carrying C2C12 (R206H) cells, compound **284** exhibited the BMP-induced alkaline phosphatase inhibitory activity with the IC_50_ value of 4.7 μM [126]. One novel oxepine-containing DPK derivative namely varioxepine B (**285**) was isolated from the fungus *Aspergillus terreus,* which was collected from soft coral *Sarcophyton subviride* on Xisha Island. Compound **285** showed excellent inhibitory activity against Con A-induced murine splenocytes with the inhibition rates of 20%, 28%, 23% and 80% at the concentration of 64, 128, 256 and 512 nM, respectively, and had no effect on cell viability at the concentration of 100 μM. Meanwhile, compound **285** also remarkably decreased the cytokine (interferon-*γ*, interleukin-2 and tumor necrosis factor-*α*) production by activating murine splenocytes. Furthermore, compound **285** showed significant inhibitory activity against anti-CD3/anti-CD28 mAb-induced murine splenocytes, human T cell proliferation and Th1/Th2 cytokine production [127].

Three novel DKP derivatives namely 3-hydroxyprotuboxepin K (**286**), 3,15-dehydroprotuboxepin K (**287**) and versiamide A (**288**) were isolated from the fungus *Aspergillus creber* EN-602 derived from marine red alga *Rhodomela confervoides* on the coast of Qingdao, China. Compound **286** showed enzyme inhibitory activity against the angiotensin converting enzyme with the IC_50_ value of 22.4 μM. In addition, compound **287** exhibited antimicrobial activities against *Edwardsiella tarda*, *E. coli*, *M. luteus*, *P. aeruginosa* and *V. harveyi* with the MIC values in the range of 8–64 μg/mL and compound **288** exhibited antimicrobial activities against *A. hydrophila*, *E. coli*, *M. luteus* and *P. aeruginosa* with the MIC values between 16 and 64 μg/mL [128]. Protuboxepins A (**289**) and B (**290**) were two novel oxepin-containing DKP derivatives and were isolated from the fungus *Aspergillus* sp. SF-5044, which was collected from the intertidal sediment from Dadaepo Beach, Busan, Korea. Compound **289** exhibited weak cytotoxic activities against HL-60, MDA-MB-231, Hep3B (human liver carcinoma), 3Y1 and K562 cell lines with the IC_50_ values of 75, 130, 150, 180 and 250 μM, respectively. Compound **289** possessed a disrupting microtubule dynamics ability and induced apoptosis in cancer because it could bind to *α*,*β*-tubulin and stabilize tubulin polymerization and then leading to chromosome misalignment and metaphase arrest in cancer [129,130].

Carnequinazolines A–C (**291**–**293**), three novel DKP derivatives, were separated from the fungus *Aspergillus carneus* KMM 4638 collected from the marine brown alga *Laminaria sachalinensis,* which was isolated from Kunachir Island. Compounds **291** and **292** had no cytotoxicity and antimicrobial activities [131]. One novel alkaloid DKP derivative namely fumiquinazoline K (**294**) was obtained from the fungus *Aspergillus fumigatus* KMM 4631, which was separated from soft coral *Sinularia* sp. in Kuril islands, and showed no enzyme inhibition and cytotoxic activities [132]. 3-[6-(2-Methylpropyl)-2-oxo-1H-pyrazin-3-yl]propanamide (**295**) was obtained from the fungus *Aspergillus versicolor* OUCMDZ-2738, and exhibited no antimicrobial and α -glucosidase inhibitory activity [107]. All 43 DKP derivatives from fungi described above are presented in Figure 8.

## 4. Characteristics of Bioactive Diketopiperazines and Their Derivatives from Marine Organisms

**Figure 9:**
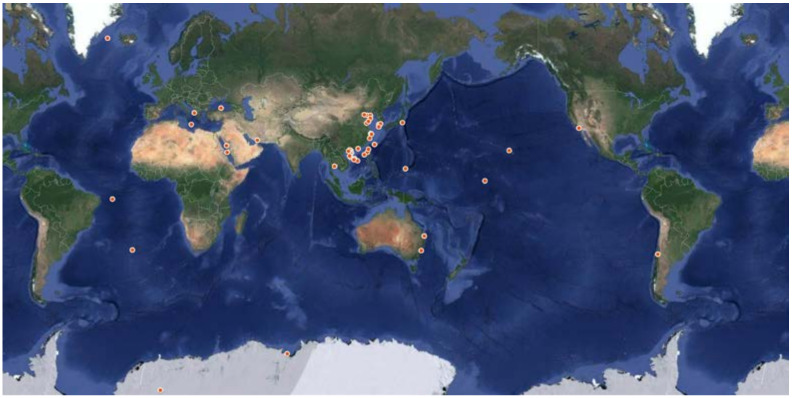
Collection points for marine biological samples producing DKPs (red dots).

**Figure 10:**
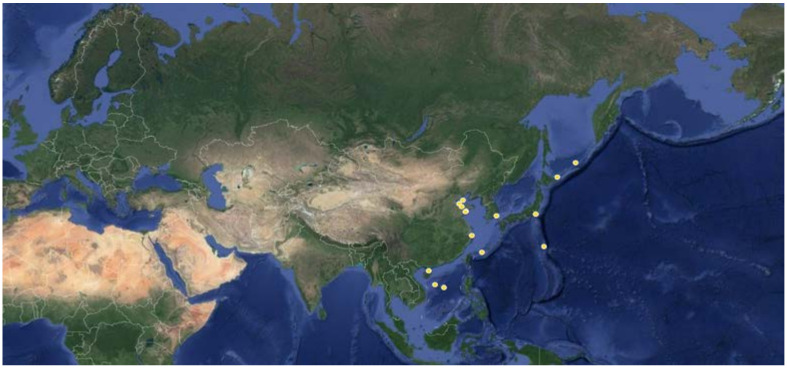
Collection points for marine biological samples producing DKP derivatives (yellow dots).

**Figure 11:**
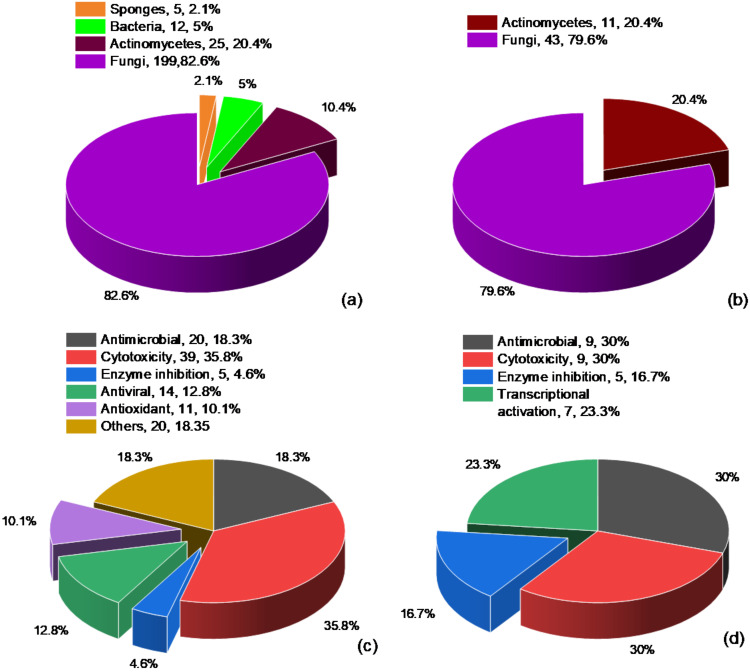
The proportion of DKPs from sponges, bacteria, actinomycetes and fungi (**a**); the proportion of DKP derivatives from actinomycetes and fungi (**b**); the proportion of antimicrobial, cytotoxicity, enzyme inhibition, antiviral, antioxidant and other activities of DKPs (**c**); the proportion of antimicrobial, cytotoxicity, enzyme inhibition and transcriptional activation activities of DKP derivatives (**d**).

In this review, 241 DKPs and 54 DKP derivatives isolated from marine organisms were summarized, among which fungi and actinomycetes were the most abundant sources. These marine organisms come from a wide range of sources and the red dots (Figure 9) and yellow dots (Figure 10) represent the collection points for marine biological samples, which produced DKPs and DKP derivatives, respectively. DKPs and DKP derivatives of fungi sources were 199 (82.6%) and 43 (79.6%), respectively, and those of actinomycetes sources were 25 (10.4%) and 11 (20.4%), respectively (Figure 11a,b). In addition, DKPs of sponge and bacteria sources were 5 (2.1%) and 12 (5%), respectively (Figure 11a).

These DKPs had antimicrobial (20, 18.3%), cytotoxic (39, 35.8%), enzyme inhibition (5, 4.6%), antiviral (14, 12.8%), antioxidant (11, 10.1%) and other activities (20, 18.3%) (Figure 11c). Furthermore, these DKP derivatives also had antimicrobial (9, 30%), cytotoxicity (9, 30%), enzyme inhibition (5, 16.7%) activities and a possessed transcriptional activation (7, 23.3%) effect (Figure 11d). Subtle differences in chemical structures are closely related to the bioactivity. For example, compound **96** possessing a unique disulfide bridge in the six-membered piperazine skeleton showed much more significant cytotoxic activity than compounds **93**–**95**, **97** and **98**. Compounds **163** and **165** exhibited remarkable antiproliferative activities but compound **164** was inactive, which indicated that the N–O bond played an important role for their bioactivity. In addition, the absolute configurations might be closely associated with the activity intensity. For instance, the antioxidant activity of compounds **144** (IC_50_ = 37 μM) and **145** (IC_50_ = 69 μM) were relevant to the absolute configurations of C-2 and C-3. Different substituents in the same chemical skeleton may lead to different activities. For example, compound **158** bearing a Cl atom at C-7 exhibited stronger antimicrobial and brine shrimp inhibition activity than compound **159**.

## 5. Conclusions

In recent years, the number of papers and patents related to DKPs is on the rise continuously and many novel DKPs had been isolated from marine sources. More and more researchers are turning their attention to the six-membered ring rigid structure with great potential for biological activity. It is a heterocyclic scaffold with restricted conformation, which can control stereochemistry at up to four positions. These features provide its potential to break the planarity of traditional drugs. Natural DKPs have more interesting structural complexity and biological characteristics and possibly can be further chemically synthesized or modified to increase their activity, promoting the natural product and synthetic chemistry to complement each other. In addition, DKPs can be used as the quorum sensing signal molecule of *Shewanella baltica* (a kind of unique microorganism produced during transporting the large yellow croaker at 4 °C) and inhibiting the production of DKPs can slow down the spoilage of the large yellow croaker [7]. Furthermore, thaxtomin A has good herbicidal activity and achieves an herbicidal purpose by inhibiting cellulose synthesis [133]. With the tremendous advancement of technology, the known compound can be initially eliminated through the Global Natural Products Social (GNPS) molecular networking project [134]. It would be helpful for targeting directly to discover new natural DKPs and further enrich the library of DKPs.

To sum up, DKPs are potential bioactive chemical substances that are valuable for further exploration from natural sources, especially from marine environments. The conversion of promising bioactive DKPs into clinical drugs for the treatment of diseases needs much more time and energy for researchers. DKPs are regarded as unprocessed diamonds, attracting scientists to take efforts to study their pharmacological properties and therapeutic effects.

## Figures and Tables

**Table 1:** The bioactivities, sources and habitats of DKPs during 2014–2021.

Sources	Compounds	Bioactivities	Species	Habitats	Refs
Sponge	Cyclo-(*R*-Pro-6-hydroxyl-*S*-Ile) (**1**)	- ^a^	*Callyspongia* sp.	South China Sea	[30]
	Geobarrettin A (**2**)	-	*G*. *barrette*	Iceland	[31]
	Geobarrettin B (**3**)	Anti-inflammatory	*G*. *barrette*	Iceland	[32]
	Tedanizaine A (**4**)	-	*Tedania* sp.	Guangdong	[32]
	(−)-Cyclo(L-*trans*-Hyp-L-Ile) (**5**)	-	*A*. *sinoxea*	Larak Island	[33]
Bacteria	Cyclo(Trp-Ser) (**6**)	Antimicrobial Antiquorum sensing	*R*. *aquimaris* QSI02	Yellow Sea	[34]
	Cyclo(Pro-Val) (**7**)	-	*G*. *antarctica* PI12	Antarctica	[35]
	(−)-Cyclo(Pro-Tyr) (**8**)	-	*G*. *antarctica* PI12	Antarctica	[35]
	(−)-Cyclo(Pro-Phe) (**9**)	-	*G*. *antarctica* PI12	Antarctica	[35]
	(+)-Cyclo(Pro-Leu) (**10**)	-	*G*. *antarctica* PI12	Antarctica	[35]
	(3*S*,6*S*)-3,6-Diisobutylpiperazine-2,5-dione (**11**)	Antimicrobial	*Bacillus* sp. SPB7	*S*. *officinalis*	[36]
	Gallaecimonamide A (**12**)	Antimicrobial	*G*. *mangrovi* HK-28	Mangrove sediment	[37]
	Gallaecimonamide A (**13**)	-	*G*. *mangrovi* HK-28	Mangrove sediment	[37]
	Gallaecimonamide A (**14**)	-	*G*. *mangrovi* HK-28	Mangrove sediment	[37]
	*cis*-Cyclo(Pro-3-chloro-Tyr) (**15**)	-	*B*. *subtilis* BI0980	Kerkyra and Erikoussa	[38]
	*trans*-Cyclo(Pro-3-chloro-Tyr) (**16**)	-	*B*. *subtilis* BI0980	Kerkyra and Erikoussa	[38]
	*cis*-Cyclo(3-chloro-Tyr-Ile) (**17**)	-	*B*. *subtilis* BI0980	Kerkyra and Erikoussa	[38]
Actinomycetes	iso-Naseseazine B (**18**)	Antimicrobial	*Streptomyces* sp. SMA-1	Yellow Sea	[39]
	Naseseazine A (**19**)	Antiplasmodial	*Streptomyces* sp. USC-636	Marine sediment	[40]
	Naseseazine B (**20**)	Antiplasmodial	*Streptomyces* sp. USC-636	Marine sediment	[40]
	Naseseazine C (**21**)	Antiplasmodial	*Streptomyces* sp. USC-636	Marine sediment	[40]
	(6*R*,3*Z*)-3-Benzylidene-6-isobutyl-1-methyl piperazine-2,5-dione (**22**)	-	*Streptomyces* sp. strain SCSIO 04496	South China Sea	[41]
	Demethylmaremycins (**23**–**28**)	-	*Streptomyces* sp. B9173	Pacific coast	[42]
	3-(3-Hydroxy-4-methoxybenzyl)-6-isobutyl-2,5-diketopiperazine (**29**)	Cytotoxicity	*Streptomyces* sp. MNU FJ-36	*Katsuwonus* sp.	[43]
	3-(1,3-Benzodioxol-5-ylmethyl)-6-isobutyl-2,5-diketopiperazine (**30**)	Cytotoxicity	*Streptomyces* sp. MNU FJ-36	*Katsuwonus* sp.	[43]
	3-(1,3-Benzodioxol-5-ylmethyl)-6-isopropyl-2,5-diketopiperazine (**31**)	Cytotoxicity	*Streptomyces* sp. MNU FJ-36	*Katsuwonus* sp.	[43]
	maculosin-*O*-*α*-L-rhamnopyranoside (**32**)	Antimicrobial	*Streptomyces* sp. ZZ446	Coastal soil	[44,45]
	Actinozine A (**33**)	Antimicrobial Cytotoxicity	*Streptomyces* species Call-36	*Callyspongia* species	[46]
	Streptodiketopiperazine A (**34**)	Antimicrobial	*Streptomyces* sp. SY1965	Mariana Trench sediment	[47]
	Streptodiketopiperazine A (**35**)	Antimicrobial	*Streptomyces* sp. SY1965	Mariana Trench sediment	[47]
	Cyclo-(4-*trans*-6-dihydroxy-proline-D-leucine) (**36**)	-	*M*. *variabilis* C-03	*Palythoa tuberculosa*	[48]
	Nocarazepine A (**37**)	-	*A*. *caerulea*	*Anthogorgia caerulea*	[49]
	Photopiperazine A (**38**)	Cytotoxicity	Strain AJS-327	Sponge fragment	[50]
	Photopiperazine B (**39**)	Cytotoxicity	Strain AJS-327	Sponge fragment	[50]
	Photopiperazine C (**40**)	Cytotoxicity	Strain AJS-327	Sponge fragment	[50]
	Photopiperazine D (41)	Cytotoxicity	Strain AJS-327	Sponge fragment	[50]
	Cyclo-(D-8-acetoxyl-Pro-L-Leu) (**42**)	Pancreatic lipase enzyme inhibition	*Treptomyces* sp. SCSIO 41400	Mangrove derived-soil	[51]
Fungus	Graphiumin A (**43**)	-	*Graphium* sp. OPMF00224	Sediment	[52]
	Graphiumin B (**44**)	-	*Graphium* sp. OPMF00224	Sediment	[52]
	Graphiumin C (**45**)	Virulence factors inhibitor	*Graphium* sp. OPMF00224	Sediment	[52]
	Graphiumin D (**46**)	Virulence factors inhibitor	*Graphium* sp. OPMF00224	Sediment	[52]
	Graphiumin E (**47**)	Virulence factors inhibitor	*Graphium* sp. OPMF00224	Sediment	[52]
	Graphiumin F (**48**)	-	*Graphium* sp. OPMF00224	Sediment	[52]
	Graphiumin G (**49**)	Virulence factors inhibitor	*Graphium* sp. OPMF00224	Sediment	[52]
	Graphiumin H (**50**)	Virulence factors inhibitor	*Graphium* sp. OPMF00224	Sediment	[52]
	Graphiumin I (**51**)	Virulence factors inhibitor	*Graphium* sp. OPMF00224	Sediment	[53]
	Graphiumin J (**52**)	Virulence factors inhibitor	*Graphium* sp. OPMF00224	Sediment	[53]
	Cladosporin A (**53**)	Cytotoxicity	*Cladosporium* sp.	Sediment	[54]
	Cladosporin B (**54**)	Cytotoxicity	*Cladosporium* sp.	Sediment	[54]
	12,13-Dihydroxy-fumitremorgin C (**55**)	-	*A*. *alternate* HK-25	Sediment	[55]
	Gliotoxin (**56**)	-	*A*. *alternate* HK-25	Sediment	[55]
	Demethoxyfumitremorgin C (**57**)	-	*A*. *alternate* HK-25	Sediment	[55]
	Bisdethiobis(methylthio)gliotoxin (**58**)	-	*A*. *alternate* HK-25	Sediment	[55]
	Fumitremorgin C (**59**)	-	*A*. *alternate* HK-25	Sediment	[55]
	Haenamindole (**60**)	Antiviral	*Penicillium* sp. KCB12F005	Sediment	[56]
	Dichotocejpin A (**61**)	Cytotoxicity *α*-glucosidase inhibitor	*D*. *cejpii* FS110	Sediment	[57,58]
	Dichotocejpin B (**62**)	-	*D*. *cejpii* FS110	Sediment	[57,58]
	Dichotocejpin C (**63**)	-	*D*. *cejpii* FS110	Sediment	[57,58]
	Cristazine (**64**)	Antioxidant Cytotoxicity	*C*. *cristatum*	Sediment	[59,60]
	Eutypellazine A (**65**)	Antiviral	*Eutypella* sp. MCCC 3A00281	Sediment	[61]
	Eutypellazine B (**66**)	Antiviral	*Eutypella* sp. MCCC 3A00281	Sediment	[61]
	Eutypellazine C (**67**)	Antiviral	*Eutypella* sp. MCCC 3A00281	Sediment	[61]
	Eutypellazine D (**68**)	Antiviral	*Eutypella* sp. MCCC 3A00281	Sediment	[61]
	Eutypellazine E (**69**)	Antiviral	*Eutypella* sp. MCCC 3A00281	Sediment	[61]
	Eutypellazine F (**70**)	Antiviral	*Eutypella* sp. MCCC 3A00281	Sediment	[61]
	Eutypellazine G (**71**)	Antiviral	*Eutypella* sp. MCCC 3A00281	Sediment	[61]
	Eutypellazine H (**72**)	Antiviral	*Eutypella* sp. MCCC 3A00281	Sediment	[61]
	Eutypellazine I (**73**)	Antiviral	*Eutypella* sp. MCCC 3A00281	Sediment	[61]
	Eutypellazine J (**74**)	Antiviral	*Eutypella* sp. MCCC 3A00281	Sediment	[61]
	Eutypellazine K (**75**)	Antiviral	*Eutypella* sp. MCCC 3A00281	Sediment	[61]
	Eutypellazine L (**76**)	Antiviral	*Eutypella* sp. MCCC 3A00281	Sediment	[61]
	Eutypellazine M (**77**)	-	*Eutypella* sp. MCCC 3A00281	Sediment	[61]
	Eutypellazine N (**78**)	-	*Eutypella* sp. MCCC 3A00281	Sediment	[62]
	Eutypellazine O (**79**)	-	*Eutypella* sp. MCCC 3A00281	Sediment	[62]
	Eutypellazine P (**80**)	-	*Eutypella* sp. MCCC 3A00281	Sediment	[62]
	Eutypellazine Q (**81**)	-	*Eutypella* sp. MCCC 3A00281	Sediment	[62]
	Eutypellazine R (**82**)	-	*Eutypella* sp. MCCC 3A00281	Sediment	[62]
	Eutypellazine S (**83**)	-	*Eutypella* sp. MCCC 3A00281	Sediment	[62]
	Fusaperazine F (**84**)	Cytotoxicity	*P*. *crustosum* HDN153086	Sediment	[63]
	Roquefortine J (**85**)	Cytotoxicity	*P*. *granulatum* MCCC 3A00475	Sediment	[64,65]
	(+)-7,8-Epoxy-brevianamide Q (**86**)	-	*A*. *versicolor* MF180151	Sediment	[66]
	(−)-7,8-Epoxy-brevianamide Q (**87**)	-	*A*. *versicolor* MF180151	Sediment	[66]
	(+)-8-Hydroxy-brevianamide R (**88**)	-	*A*. *versicolor* MF180151	Sediment	[66]
	(−)-8-Hydroxy-brevianamide R (**89**)	-	*A*. *versicolor* MF180151	Sediment	[66]
	(+)-8-Epihydroxy-brevianamide R (**90**)	-	*A*. *versicolor* MF180151	Sediment	[66]
	(−)-8-Epihydroxy-brevianamide R (**91**)	-	*A*. *versicolor* MF180151	Sediment	[66]
	Raistrickindole A (**92**)	Antiviral	*P*. *raistrickii* IMB17-034	Sediment	[67]
	5′-Hydroxy-6′-ene-epicoccin G (**93**)	-	*E*. *nigrum* SD-388	Sediment	[68]
	7-Methoxy-7′-hydroxyepicoccin G (**94**)	-	*E*. *nigrum* SD-388	Sediment	[68]
	8′-Acetoxyepicoccin D (**95**)	-	*E*. *nigrum* SD-388	Sediment	[68]
	7′-Demethoxyrostratin C (**96**)	Cytotoxicity	*E*. *nigrum* SD-388	Sediment	[68]
	(+)-5-hydroxydiphenylalazine A (**97**)	-	*E*. *nigrum* SD-388	Sediment	[68]
	(−)-5-hydroxydiphenylalazine A (**98**)	-	*E*. *nigrum* SD-388	Sediment	[68]
	(+) Eurotinoid A (**99**)	Antioxidant	*Eurotium* sp. SCSIO F452	Sediment	[69]
	(−) Eurotinoid A (**100**)	Antioxidant	*Eurotium* sp. SCSIO F452	Sediment	[69]
	(+) Eurotinoid B (**101**)	Antioxidant	*Eurotium* sp. SCSIO F452	Sediment	[69]
	(−) Eurotinoid B (**102**)	Antioxidant	*Eurotium* sp. SCSIO F452	Sediment	[69]
	(+) Eurotinoid C (**103**)	Antioxidant	*Eurotium* sp. SCSIO F452	Sediment	[69]
	(−) Eurotinoid C (**104**)	Antioxidant	*Eurotium* sp. SCSIO F452	Sediment	[69]
	Versicamide A (**105**)	-	*A*. *versicolor* HDN08-60	Sediment	[70]
	Versicamide B (**106**)	-	*A*. *versicolor* HDN08-60	Sediment	[70]
	Versicamide C (**107**)	-	*A*. *versicolor* HDN08-60	Sediment	[70]
	Versicamide D (**108**)	-	*A*. *versicolor* HDN08-60	Sediment	[70]
	Versicamide E (**109**)	-	*A*. *versicolor* HDN08-60	Sediment	[70]
	Versicamide F (**110**)	-	*A*. *versicolor* HDN08-60	Sediment	[70]
	Versicamide G (**111**)	-	*A*. *versicolor* HDN08-60	Sediment	[70]
	Versicamide H (**112**)	Cytotoxicity	*A*. *versicolor* HDN08-60	Sediment	[70]
	Rubrumline A (**113**)	-	*E*. *rubrum*	Sediment	[71]
	Rubrumline B (**114**)	-	*E*. *rubrum*	Sediment	[71]
	Rubrumline C (**115**)	-	*E*. *rubrum*	Sediment	[71]
	Rubrumline D (**116**)	Antiviral	*E*. *rubrum*	Sediment	[71]
	Rubrumline E (**117**)	-	*E*. *rubrum*	Sediment	[71]
	Rubrumline F (**118**)	-	*E*. *rubrum*	Sediment	[71]
	Rubrumline G (**119**)	-	*E*. *rubrum*	Sediment	[71]
	Rubrumline H (**120**)	-	*E*. *rubrum*	Sediment	[71]
	Rubrumline I (**121**)	-	*E*. *rubrum*	Sediment	[71]
	Rubrumline J (**122**)	-	*E*. *rubrum*	Sediment	[71]
	Rubrumline K (**123**)	-	*E*. *rubrum*	Sediment	[71]
	Rubrumline L (**124**)	-	*E*. *rubrum*	Sediment	[71]
	Rubrumline M (**125**)	-	*E*. *rubrum*	Sediment	[71]
	Rubrumline N (**126**)	-	*E*. *rubrum*	Sediment	[71]
	Rubrumline O (**127**)	-	*E*. *rubrum*	Sediment	[71]
	12*β*-Hydroxy-13*α*-ethoxyverruculogen TR-2 (**128**)	-	*Penicillium* sp. DT-F29	Sediment	[72]
	12*β*-Hydroxy-13*α*-butoxyethoxyverruculogen TR-2 (**129**)	-	*Penicillium* sp. DT-F29	Sediment	[72]
	Hydrocycloprostatin A (**130**)	-	*Penicillium* sp. DT-F29	Sediment	[72]
	Hydrocycloprostatin B (**131**)	-	*Penicillium* sp. DT-F29	Sediment	[72]
	25-Hydroxyfumitremorgin B (**132**)	-	*Penicillium* sp. DT-F29	Sediment	[72]
	12*β*-Hydroxy-13*α*-butoxyethoxyfumitremorgin B (**133**)	-	*Penicillium* sp. DT-F29	Sediment	[72]
	12*β*-Hydroxy-13*α*-methoxyverruculogen (**134**)	BRD4 protein inhibition	*Penicillium* sp. DT-F29	Sediment	[72]
	26*α*-Hydroxyfumitremorgin A (**135**)	-	*Penicillium* sp. DT-F29	Sediment	[72]
	25-Hydroxyfumitremorgin A (**136**)	-	*Penicillium* sp. DT-F29	Sediment	[72]
	Diprostatin A (**137**)	BRD4 protein inhibition	*Penicillium* sp. DT-F29	Sediment	[72]
	(+) variecolortin A (**138**)	Antioxidant	*Eurotium* sp. SCSIO F452	Sediment	[73]
	(−) variecolortin A (**139**)	-	*Eurotium* sp. SCSIO F452	Sediment	[73]
	(+) variecolortin B (**140**)	Cytotoxicity	*Eurotium* sp. SCSIO F452	Sediment	[73]
	(−) variecolortin B (**141**)	-	*Eurotium* sp. SCSIO F452	Sediment	[73]
	(+) variecolortin C (**142**)	Cytotoxicity	*Eurotium* sp. SCSIO F452	Sediment	[73]
	(−) variecolortin C (**143**)	-	*Eurotium* sp. SCSIO F452	Sediment	[73]
	Eurotiumin A (**144**)	Antioxidant	*Eurotium* sp. SCSIO F452	Sediment	[74]
	Eurotiumin B (**145**)	Antioxidant	*Eurotium* sp. SCSIO F452	Sediment	[74]
	Eurotiumin C (**146**)	Antioxidant	*Eurotium* sp. SCSIO F452	Sediment	[74]
	6-Acetylmonodethiogliotoxin (**147**)	NF-*κ*B inhibitor	*D*. *cejpii*	*Callyspongia* cf. *C*. *flammea*	[75]
	6-Acetylbisdethiobis(methylthio)gliotoxin (**148**)	NF-*κ*B inhibitor	*D*. *cejpii*	*Callyspongia* cf. *C*. *flammea*	[75]
	5a,6-Anhydrobisdethiobis(methyl-thio)gliotoxin (**149**)	NF-*κ*B inhibitor	*D*. *cejpii*	*Callyspongia* cf. *C*. *flammea*	[75]
	Peniciadametizine A (**150**)	Antimicrobial	*P*. *adametzioides* AS-53	Unidentified sponge	[76]
	peniciadametizine B (**151**)	Antimicrobial	*P*. *adametzioides* AS-53	Unidentified sponge	[76]
	Cyclo-(2-hydroxy-Pro-Gly) (**152**)	-	*Simplicillium* sp. YZ-11	*H*. *perleve*	[77]
	Fellutanine A (**153**)	-	*N*. *glabra* KUFA 0702	*Mycale* sp.	[78]
	Asperflocin (**154**)	Cytotoxicity	*A*. *versicolor* 16F-11	*P*. *fusca*	[79]
	Unnamed diketopiperazine dimer (**155**)	Anti-inflammatory	*A*. *violaceofuscus*	*Reniochalina* sp.	[80]
	Waspergillamide B (**156**)	-	*A*. *ochraceus*	*A*. *oroides*	[81]
	Penicillivinacine (**157**)	Cytotoxicity	*P*. *vinaceum*	*H*. *erectus*	[82]
	Adametizine A (**158**)	Antimicrobial Brine shrimp lethality	*P*. *adametzioides* AS-53	unidentified sponge	[83]
	Adametizine B (**159**)	Antimicrobial Brine shrimp lethality	*P*. *adametzioides* AS-53	unidentified sponge	[83]
	SF5280-415 (**160**)	Enzyme inhibition	*Aspergillus* sp. SF-5280	unidentified sponge	[84]
	Penicimutide (**161**)	Cytotoxicity	*P*. *purpurogenum* G59	Bohai Bay	[85]
	Penicimutanin C (**162**)	Cytotoxicity	*P*. *purpurogenum* G59	Bohai Bay	[86]
	Waikikiamide A (**163**)	Cytotoxicity	*Aspergillus* sp. FM242	Waikiki beach	[87]
	Waikikiamide B (**164**)	-	*Aspergillus* sp. FM242	Waikiki beach	[87]
	Waikikiamide C (**165**)	Cytotoxicity	*Aspergillus* sp. FM242	Waikiki beach	[87]
	5*S*-Hydroxynorvaline-*S*-Ile (**166**)	-	*Penicillium* sp. GD6	*B*. *gymnorrhiza*	[88]
	3*S*-Hydroxylcyclo(*S*-Pro-*S*-Phe) (**167**)	-	*Penicillium* sp. GD6	*B*. *gymnorrhiza*	[88]
	Cyclo(*S*-Phe-*S*-Gln) (**168**)	-	*Penicillium* sp. GD6	*B*. *gymnorrhiza*	[88]
	(−)-Asperginulin A (**169**)	-	*Aspergillus* sp. SK-28	*K*. *candel*	[89]
	(+)-Asperginulin A (**170**)	Antifouling	*Aspergillus* sp. SK-28	*K*. *candel*	[89]
	Saroclazine A (**171**)	Cytotoxicity	*S*. *kiliense* HDN11-84	*T*. *populnea*	[90]
	Saroclazine B (**172**)	-	*S*. *kiliense* HDN11-84	*T*. *populnea*	[90]
	Saroclazine C (**173**)	-	*S*. *kiliense* HDN11-84	*T*. *populnea*	[90]
	Brocazine A (**174**)	Cytotoxicity	*P*. *brocae* MA-231	*A*. *marina*	[91]
	Brocazine B (**175**)	Cytotoxicity	*P*. *brocae* MA-231	*A*. *marina*	[91]
	Brocazine C (**176**)	Cytotoxicity	*P*. *brocae* MA-231	*A*. *marina*	[91]
	Brocazine D (**177**)	Cytotoxicity	*P*. *brocae* MA-231	*A*. *marina*	[91]
	Brocazine E (**178**)	-	*P*. *brocae* MA-231	*A*. *marina*	[91]
	Brocazine F (**179**)	-	*P*. *brocae* MA-231	*A*. *marina*	[91]
	Penicibrocazine A (**180**)	-	*P*. *brocae* MA-231	*A*. *marina*	[92]
	Penicibrocazine B (**181**)	-	*P*. *brocae* MA-231	*A*. *marina*	[92]
	Penicibrocazine C (**182**)	Antimicrobial	*P*. *brocae* MA-231	*A*. *marina*	[92]
	Penicibrocazine D (**183**)	-	*P*. *brocae* MA-231	*A*. *marina*	[92]
	Penicibrocazine E (**184**)	Antimicrobial	*P*. *brocae* MA-231	*A*. *marina*	[92]
	Spirobrocazine A (**185**)	Antimicrobial	*P*. *brocae* MA-231	*A*. *marina*	[93]
	Spirobrocazine B (**186**)	-	*P*. *brocae* MA-231	*A*. *marina*	[93]
	Spirobrocazine C (**187**)	Antimicrobial	*P*. *brocae* MA-231	*A*. *marina*	[93]
	Brocazine G (**188**)	Antimicrobial Cytotoxicity	*P*. *brocae* MA-231	*A*. *marina*	[93]
	Pseudellone A (**189**)	-	*P*. *ellipsoidea* F42−3	*L*. *crassum*	[94]
	Pseudellone B (**190**)	-	*P*. *ellipsoidea* F42−3	*L*. *crassum*	[94]
	Pseuboydone C (**191**)	Cytotoxicity	*P*. *boydii* F19-1	*L*. *crassum*	[95]
	Pseuboydone D (**192**)	-	*P*. *boydii* F19-1	*L*. *crassum*	[95]
	Dichocerazine A (**193**)	-	*D*. *cejpii* F31-1	*L*. *crassum*	[96]
	Dichocerazine B (**194**)	-	*D*. *cejpii* F31-1	*L*. *crassum*	[96]
	11-Methylneoechinulin E (**195**)	Cytotoxicity	*Aspergillus* sp. EGF 15-0-3	South China Sea	[97]
	Variecolorin M (**196**)	Cytotoxicity	*Aspergillus* sp. EGF 15-0-3	South China Sea	[97]
	(+)-Variecolorin G (**197**)	Cytotoxicity	*Aspergillus* sp. EGF 15-0-3	South China Sea	[97]
	(+)-Neoechinulin A (**198**)	Cytotoxicity	*Aspergillus* sp. EGF 15-0-3	South China Sea	[97]
	16α-Hydroxy-17β-methoxy-deoxydihydroisoaustamide (**199**)	-	*P*. *dimorphosporum* KMM 4689	South China Sea	[98]
	16β-Hydroxy-17α-methoxy-deoxydihydroisoaustamide (**200**)	-	*P*. *dimorphosporum* KMM 4689	South China Sea	[98]
	16β,17α-dihydroxy-deoxydihydroisoaustamide (**201**)	-	*P*. *dimorphosporum* KMM 4689	South China Sea	[98]
	16α-hydroxy-17α-methoxy-deoxydihydroisoaustamide (**202**)	Enhance cell viability	*P*. *dimorphosporum* KMM 4689	South China Sea	[98]
	16α,17α-dihydroxy-deoxydihydroisoaustamide (**203**)	Enhance cell viability	*P*. *dimorphosporum* KMM 4689	South China Sea	[98]
	16,17-dihydroxy-deoxydihydroisoaustamide (**204**)	Enhance cell viability	*P*. *dimorphosporum* KMM 4689	South China Sea	[98]
	3β-Hydroxy-deoxyisoaustamide (**205**)	-	*P*. *dimorphosporum* KMM 4689	South China Sea	[98]
	Pseudellone D (206)	-	*P*. *ellipsoidea* F42-3	*L*. *crissum*	[99]
	Dehydroxymethylbis(dethio)bis(methylthio)gliotoxin (**207**)	-	*T*. *virens* Y13-3	*G*. *vermiculoph-ylla*	[100]
	(3*S*,6*R*)-6-(Para-hydroxybenzyl)-1,4-dimethyl-3,6-bis(methylthio)pip-erazine-2,5-dione (**208**)	-	*T*. *virens* Y13-3	*G*. *vermiculoph-ylla*	[100]
	Methylcordysinin A (**209**)	-	*T*. *asperellum* cf44-2	*Sargassum* sp.	[101]
	Citriperazine A (**210**)	-	*Penicillium* sp. KMM 4672	*Padina* sp.	[102]
	Citriperazine B (**211**)	-	*Penicillium* sp. KMM 4672	*Padina* sp.	[102]
	Citriperazine C (**212**)	-	*Penicillium* sp. KMM 4672	*Padina* sp.	[102]
	Citriperazine D (**213**)	-	*Penicillium* sp. KMM 4672	*Padina* sp.	[102]
	(+) Acrozines A (**214**)	Antiacetylcholinesterase	*A*. *luteoalbus* TK-43	*C*. *fragile*	[103]
	(−) Acrozines A (**215**)	Antiacetylcholinesterase	*A*. *luteoalbus* TK-43	*C*. *fragile*	[103]
	(+) Acrozines B (**216**)	Antimicrobial	*A*. *luteoalbus* TK-43	*C*. *fragile*	[103]
	(−) Acrozines B (**217**)	-	*A*. *luteoalbus* TK-43	*C*. *fragile*	[103]
	(+) Acrozines C (**218**)	-	*A*. *luteoalbus* TK-43	*C*. *fragile*	[103]
	(−) Acrozines C (**219**)	-	*A*. *luteoalbus* TK-43	*C*. *fragile*	[103]
	Cyclo(L-5-MeO-Pro-L-5-MeO-Pro) (**220**)	Antimicrobial	*T*. *asperellum* A-YMD-9-2	*G*. *verrucose*	[104]
	Pretrichodermamide D (**221**)	-	*Penicillium* sp. KMM 4672	*Padina* sp.	[105]
	Pretrichodermamide E (**222**)	-	*Penicillium* sp. KMM 4672	*Padina* sp.	[105]
	Pretrichodermamide F (**223**)	-	*Penicillium* sp. KMM 4672	*Padina* sp.	[105]
	N-(4′-hydroxyprenyl)-cyclo(alanyltryptophyl) (**224**)	-	*E*. *cristatum* EN-220	*S*. *thunbergia*	[106]
	Isovariecolorin I (**225**)	Brine shrimp lethal	*E*. *cristatum* EN-220	*S*. *thunbergia*	[106]
	30-Hydroxyechinulin (**226**)	-	*E*. *cristatum* EN-220	*S*. *thunbergia*	[106]
	29-Hydroxyechinulin (**227**)	-	*E*. *cristatum* EN-220	*S*. *thunbergia*	[106]
	(+)-Brevianamide X (**228**)	-	*A*. *versicolor* OUCMDZ-2738	*E*. *prolifera*	[107]
	(−)-Brevianamide X (**229**)	-	*A*. *versicolor* OUCMDZ-2738	*E*. *prolifera*	[107]
	Isoechinulin D (**230**)	-	*E*. *rubrum* MPUC136	/ ^b^	[108]
	Spirotryprostatin G (**231**)	Cytotoxicity	*P*. *brasilianum* HBU-136	Bohai Sea	[109]
	Cyclotryprostatin F (**232**)	Cytotoxicity	*P*. *brasilianum* HBU-136	Bohai Sea	[109]
	Cyclotryprostatin G (**233**)	Cytotoxicity	*P*. *brasilianum* HBU-136	Bohai Sea	[109]
	Penilline C (**234**)	-	*P*. *chrysogenum* SCSIO 07007	Western Atlantic	[110]
	Emestrin L (**235**)	-	*A*. *terreus* RA2905	*A*. *pulmonica*	[111]
	Emestrin M (**236**)	Antimicrobial	*A*. *terreus* RA2905	*A*. *pulmonica*	[111]
	Aspamide A (**237**)	-	*A*. *versicolor* DY180635	*C*. *haematocheir*	[112]
	Aspamide B (**238**)	-	*A*. *versicolor* DY180635	*C*. *haematocheir*	[112]
	Aspamide C (**239**)	-	*A*. *versicolor* DY180635	*C*. *haematocheir*	[112]
	Aspamide D (**240**)	-	*A*. *versicolor* DY180635	*C*. *haematocheir*	[112]
	Penicillatide B (**241**)	Cytotoxicity Antimicrobial	*Penicillium* sp.	*Didemnum* sp.	[113]

^a^ The bioactivity was not mentioned; ^b^ the habitat was not mentioned.

**Table 2:** The bioactivities, strains and habitats of diketopiperazine derivatives during 2011–2021.

Sources	Compounds	Bioactivities	Species	Habitats	Refs
Actinomycetes	Isomethoxyneihumicin (**242** and **243**)	Cytotoxicity	*N*. *alba* KM6-1	Marine sediment	[114]
	Nocazine F (**244**)	Cytotoxicity	*Nocardiopsis* sp. YIM M13066	Deep-sea sediment	[115]
	Nocazine G (**245**)	Cytotoxicity Antimicrobial	*Nocardiopsis* sp. YIM M13066	Deep-sea sediment	[115]
	Streptopyrazinone A (**246**)	Antimicrobial	*Streptomyces* sp. ZZ446	Coastal soil	[44]
	Streptopyrazinone B (**247**)	Antimicrobial	*Streptomyces* sp. ZZ446	Coastal soil	[44]
	Streptopyrazinone C (**248**)	Antimicrobial	*Streptomyces* sp. ZZ446	Coastal soil	[44]
	Streptopyrazinone D (**249**)	Antimicrobial	*Streptomyces* sp. ZZ446	Coastal soil	[44]
	Streptopyrazinone (**250**)	Cytotoxicity	*Streptomyces* sp. B223	Marine sediment	[116]
	Nocazine A (**251**)	-	*N*. *dassonvillei* HR10-5	Estuary of Yellow River	[117]
	Nocazine B (**252**)	-	*N*. *dassonvillei* HR10-5	Estuary of Yellow River	[117]
Fungus	Varioloid A (**253**)	Antimicrobial	*P*. *variotii* EN-291	*G*. *turuturu*	[118]
	Varioloid B (**254**)	Antimicrobial	*P*. *variotii* EN-291	*G*. *turuturu*	[118]
	Oxepinamide H (**255**)	Transcriptional activation	*A*. *puniceus* SCSIO z021	Deep-sea sediment	[119]
	Oxepinamide I (**256**)	Transcriptional activation	*A*. *puniceus* SCSIO z021	Deep-sea sediment	[119]
	Oxepinamide J (**257**)	Transcriptional activation	*A*. *puniceus* SCSIO z021	Deep-sea sediment	[119]
	Oxepinamide K (**258**)	-	*A*. *puniceus* SCSIO z021	Deep-sea sediment	[119]
	Puniceloid A (**259**)	Transcriptional activation	*A*. *puniceus* SCSIO z021	Deep-sea sediment	[119]
	Puniceloid B (**260**)	Transcriptional activation	*A*. *puniceus* SCSIO z021	Deep-sea sediment	[119]
	Puniceloid C (**261**)	Transcriptional activation	*A*. *puniceus* SCSIO z021	Deep-sea sediment	[119]
	Puniceloid D (**262**)	Transcriptional activation Enzyme inhibition	*A*. *puniceus* SCSIO z021	Deep-sea sediment	[119]
	Protuboxepin C (**263**)	Cytotoxicity	*Aspergillus* sp. SCSIO XWS02F40	*Callyspongia* sp.	[120,121]
	Protuboxepin D (**264**)	Cytotoxicity	*Aspergillus* sp. SCSIO XWS02F40	*Callyspongia* sp.	[120,121]
	Pyranamide A (**265**)	-	*A*. *versicolor* SCSIO 41016	Sponge	[122]
	Pyranamide A (**266**)	-	*A*. *versicolor* SCSIO 41016	Sponge	[122]
	Pyranamide A (**267**)	-	*A*. *versicolor* SCSIO 41016	Sponge	[122]
	Pyranamide A (**268**)	-	*A*. *versicolor* SCSIO 41016	Sponge	[122]
	Secopyranamide C (**269**)	-	*A*. *versicolor* SCSIO 41016	Sponge	[122]
	Protuboxepin F (**270**)	Cytotoxicity	*A*. *versicolor* SCSIO 41016	Sponge	[122]
	Protuboxepin G (**271**)	-	*A*. *versicolor* SCSIO 41016	Sponge	[122]
	Protuboxepin H (**272**)	-	*A*. *versicolor* SCSIO 41016	Sponge	[122]
	Protuboxepin I (**273**)	-	*A*. *versicolor* SCSIO 41016	Sponge	[122]
	Protuboxepin J (**274**)	-	*A*. *versicolor* SCSIO 41016	Sponge	[122]
	Chrysopiperazine A (**275**)	-	*P*. *chrysogenum*	*D*. *gemmacea*	[123]
	Chrysopiperazine B (**276**)	-	*P*. *chrysogenum*	*D*. *gemmacea*	[123]
	Chrysopiperazine C (**277**)	-	*P*. *chrysogenum*	*D*. *gemmacea*	[123]
	Quinadoline D (**278**)	-	*Penicillium* sp. L129	*L*. *sinense*	[124]
	Aspamide F (**279**)	-	*A*. *versicolor* DY180635	*C*. *haematocheir*	[110]
	Aspamide G (**280**)	-	*A*. *versicolor* DY180635	*C*. *haematocheir*	[110]
	Polonimide A (**281**)	Enzyme inhibition	*P*. *polonicum*	Bohai Sea	[125]
	Polonimide B (**282**)	Enzyme inhibition	*P*. *polonicum*	Bohai Sea	[125]
	Polonimide C (**283**)	Enzyme inhibition	*P*. *polonicum*	Bohai Sea	[125]
	Protuboxepin K (**284**)	Enzyme inhibition	*Aspergillus* sp. BFM-0085	Marine sediment	[126]
	Varioxepine B (**285**)	Cytotoxicity	*A*. *terreus*	*S*. *subviride*	[127]
	3-Hydroxyprotuboxepin K (**286**)	Enzyme inhibition	*A*. *creber* EN-602	*R*. *confervoides*	[128]
	3,15-Hehydroprotuboxepin K (**287**)	Antimicrobial	*A*. *creber* EN-602	*R*. *confervoides*	[128]
	Versiamide A (**288**)	Antimicrobial	*A*. *creber* EN-602	*R*. *confervoides*	[128]
	Protuboxepin A (**289**)	Cytotoxicity	*Aspergillus* sp. SF-5044	Sediment	[129,130]
	Protuboxepin B (**290**)	-	*Aspergillus* sp. SF-5044	Sediment	[129,130]
	Carnequinazoline A (**291**)	-	*A*. *carneus* KMM 4638	*L*. *sachalinensis*	[131]
	Carnequinazoline B (**292**)	-	*A*. *carneus* KMM 4638	*L*. *sachalinensis*	[131]
	Carnequinazoline C (**293**)	-	*A*. *carneus* KMM 4638	*L*. *sachalinensis*	[131]
	Fumiquinazoline K (**294**)	-	*A*. *fumigatus* KMM 4631	*Sinularia* sp.	[132]
	3-[6-(2-Methylpropyl)-2-oxo-1H-pyrazin-3-yl]propanamide (**295**)	-	*A*. *versicolor* OUCMDZ-2738	*E*. *prolifera*	[107]

^a^ The bioactivity was not mentioned.

## Data Availability

All data in this article is openly available without any restrictions.
